# DLBCL-microenvironment interactions: cytokine profiling and ECM-mediated ibrutinib resistance in a 3D bone-based model

**DOI:** 10.1007/s12032-026-03309-3

**Published:** 2026-07-15

**Authors:** Jessica Ceccato, Maria Piazza, Giulia Gualtiero, Samuela Carraro, Francesco Cinetto, Carlo Biz, Sabrina Manni, Marco Pizzi, Sabrina Pianalto, Simone Zoletto, Valeria Carabotta, Nicolo’ Danesin, Pietro Ruggieri, Angelo Paolo Dei Tos, Francesco Piazza, Livio Trentin, Fabrizio Vianello

**Affiliations:** 1 Hematology Unit, Department of Medicine–DIMED, University-Hospital of Padua, Padua, Italy; 2 Laboratory of Myeloma and Lymphoma Pathobiology, Veneto Institute of Molecular Medicine (VIMM), Padua, Italy; 3 Internal Medicine and Allergology and Clinical Immunology Unit, Treviso Ca’ Foncello Hospital, Treviso, Italy; 4 Orthopedics and Orthopedic Oncology, Department of Surgery, Oncology and Gastroenterology (DiSCOG), University-Hospital of Padua, Padua, Italy; 5 Pathology Unit, Department of Medicine-DIMED, University-Hospital of Padua, Padua, Italy

**Keywords:** Diffuse large B cell lymphoma, Organoid, Scaffold, Ibrutinib, Extracellular matrix

## Abstract

Diffuse large B-cell lymphoma (DLBCL) frequently relapses following therapy, partly due to microenvironment-mediated drug resistance. Bone marrow involvement is associated with poor prognosis, yet preclinical models that faithfully recapitulate its extracellular matrix (ECM) remain limited. We used a decellularized human bone-derived 3D scaffold to evaluate the impact of extracellular matrix (ECM) interactions on proliferation, cytokine secretion, and ibrutinib sensitivity in four DLBCL cell lines (OCI-LY1, OCI-LY18, RIVA, NU-DUL-1). Functional assays and cytokine profiling were performed under 2D and 3D culture conditions. Compared with 2D cultures, the 3D ECM model induced a profound remodeling of the DLBCL secretome, with upregulation of a coordinated network of pro-migratory chemokines, particularly CXCL9, CCL22, CCL17, CCL4 and CXCL1/2/3. ECM engagement enhanced DLBCL migration and promoted scaffold colonization, indicating a positive feedback loop between tumor cells and the microenvironment. While all DLBCL cell lines were sensitive to ibrutinib in 2D, ECM-adherent OCI-LY18 and RIVA cells showed reduced drug-induced apoptosis in 3D. This effect was dependent on direct ECM contact, was not reproduced by inert 3D scaffold, and was fully reversible upon disruption of cell-ECM interaction. Mechanistically, ECM adhesion was associated with activation of the AKT/mTOR pathway. The human bone-derived 3D ECM model reveals that direct tumor-matrix interactions induce reversible ibrutinib resistance and reshape the cytokine milieu in DLBCL. These findings highlight the role of ECM as a dynamic regulator of drug response and support the use of physiologically relevant 3D models to investigate microenvironment-driven resistance and guide therapeutic strategies.

**Supplementary Information:**

The online version contains supplementary material available at https://doi.org/10.1007/s12032-026-03309-3.

## Background

Diffuse large B-cell lymphoma (DLBCL) is the most common subtype of non-Hodgkin lymphoma (NHL), accounting for nearly 30% of cases worldwide [1]. Most cases arise de novo*,* although some evolve from pre-existing lymphoproliferative disorders [2]. The introduction of rituximab in combination with CHOP chemotherapy significantly improved outcomes, establishing R-CHOP as the standard first-line treatment, with remission rates approaching 70%. However, up to one-third of patients relapse or are refractory to therapy, highlighting the need to better understand mechanisms of treatment resistance [1].

Bruton’s tyrosine kinase (BTK) inhibitors, such as ibrutinib, have emerged as promising agents targeting B-cell receptor signaling (BCR) [3]. However, clinical responses remain heterogeneous, particularly in patients with bone marrow (BM) involvement, which occurs in approximately 30% of cases and is associated with poor prognosis. This suggests that interactions between tumor cells and the bone marrow microenvironment may contribute to treatment resistance.

Conventional two-dimensional (2D) cultures fail to capture these microenvironmental cues, while three-dimensional (3D) spheroid models, although useful for cell–cell studies, do not reproduce the architectural and biochemical complexity of the ECM [4, 5]. To address these limitations, we employed a human bone-derived decellularized scaffold, previously developed and validated by our group, that preserves the native three-dimensional architecture, biochemical composition, and biophysical properties of the bone marrow ECM.

This platform enables the study of direct DLBCL–ECM interactions in a physiologically relevant setting that closely mimics the bone marrow niche. It therefore provides a translational model to investigate ECM-mediated mechanisms of drug resistance and to explore how the microenvironment shapes cytokine secretion, migration, and therapeutic response in bone marrow–infiltrating DLBCL [6].

## Methods

This study was approved by the Ethics Committee of Azienda Ospedaliera di Padova (Approval No 1854). All methods were carried out in accordance with relevant guidelines and regulations. Informed consent was obtained from all participants before their involvement in the study.

### Cell lines and culture conditions

All cell lines and 3D models were maintained at 37°C in a humidified atmosphere with 5% CO₂. Four DLBCL cell lines were used: two activated B-cell–like (ABC) lines, NU-DUL-1 and RIVA, and two germinal center B-cell–like (GCB) lines, OCI-LY18 and OCI-LY1 (DSMZ, Germany). OCI-LY18, RIVA, and NU-DUL-1 cells were cultured in RPMI 1640 medium (Euroclone, Italy) supplemented with 1% penicillin/streptomycin, 0.05 mM 2-mercaptoethanol, and 10% fetal bovine serum (FBS), except for NU-DUL-1, which was maintained in 20% FBS. OCI-LY1 cells were cultured in IMDM (Euroclone, Italy) supplemented with 1% penicillin/streptomycin, 0.05 mM 2-mercaptoethanol, and 20% FBS.

### Preparation of decellularized bone ECM scaffolds

Human femoral bone samples were obtained from patients undergoing orthopedic surgery at the University Hospital of Padua after written informed consent. Bone fragments were processed into standardized specimens measuring 2 × 2 × 1 mm (length × depth × thickness) and decellularized using a previously validated protocol, as already described [7].

### Establishment of the 3D ECM-DLBCL model

To generate the 3D ECM-DLBCL model, 0.5 × 10⁶ lymphoma cells (OCI-LY18, OCI-LY1, NU-DUL-1, or RIVA) were resuspended in 1 mL of culture medium and seeded onto individual ECM scaffolds placed in 24-well plates. During culture, a fraction of cells spontaneously adhered to the scaffold and established the 3D culture, whereas non-adherent cells settled at the bottom of the well (Supplementary Fig. 1a). When required, bone marrow mesenchymal stromal cells (BM-MSCs) were isolated from bone marrow aspirates without lymphoma infiltration and used for scaffold recellularization, as described in the Supplementary Methods.

### Drug treatment

For drug treatment experiments, 3D models were first established as described above and maintained for 72 h to allow lymphoma cell adhesion and stabilization within the scaffold [7]. After this incubation period, ibrutinib (Selleckchem, USA) was added for 24 h at the following final concentrations: 10 μM for OCI-LY18, 40 μM for RIVA, and 50 μM for OCI-LY1 and NU-DUL-1. Doxorubicin (Selleckchem, USA) was administered at a final concentration of 1 μM to OCI-LY18 and primed OCI-LY18 cells. The selected drug concentrations were defined on the basis of preliminary dose–response experiments aimed at obtaining measurable apoptotic responses within a fixed exposure time in vitro, particularly under 3D ECM-based conditions, where reduced drug diffusion and microenvironment-mediated protection may attenuate apparent drug sensitivity. At the end of treatment, cells were collected for downstream analyses. In 3D conditions, both scaffold-adherent and non-adherent fractions were included in the evaluation.

### Generation of primed OCI-LY18 cells

Primed OCI-LY18 cells were generated from OCI-LY18 cells that had adhered to ECM scaffolds in three independent 3D ECM-DLBCL cultures and were subsequently re-expanded under 2D conditions. The detailed procedure is reported in the Supplementary Methods and summarized in Supplementary Fig. 2.

### Migration assay

The chemoattractive effect exerted by the 3D model or by conditioned medium was assessed by transwell migration assay. Polycarbonate transwell inserts for 24-well plates (6.5 mm diameter, 5 μm pore size; Corning, USA) were used. For each conditions, 600 μL of fresh culture medium or conditioned medium were placed in the lower chamber. OCI-LY18 cells were stained with 125 μM SYTO™ 16 Green Fluorescent Nucleic Acid Stain (Thermo Fisher, USA) in Hanks’ Balanced Salt Solution (HBSS; Gibco, USA) for 20 min in the dark. After staining, 1 × 10⁶ OCI-LY18 cells were resuspended in 200 μL of culture medium and added to the upper chamber (Supplementary Fig. 1b). After 4 h of incubation, the entire volume from the lower chamber (600 μL) was collected and analyzed by flow cytometry. Migration induced by conditioned medium and by the complete 3D model was compared with migration observed in the presence of scaffold alone or fresh medium alone. To investigate whether the chemoattractive effect depended on soluble factors present in the conditioned medium, conditioned medium was diluted 1:2 or 1:5 with fresh medium before use. In separate experiments, conditioned medium was also added to the upper chamber in order to abolish the concentration gradient.

### Scaffold colonization assay

To assess whether previously established 3D-adherent cells could promote further scaffold colonization, a secondary colonization assay was performed. First, 0.5 × 10⁶ OCI-LY18 cells were cultured on ECM scaffolds for 72 h to generate the 3D ECM-DLBCL model. Subsequently, each scaffold was re-seeded with 0.5 × 10⁶ SYTO-labeled OCI-LY18 cells. As control conditions, ECM scaffolds were also seeded with pre-stained OCI-LY18 cells in the presence of either fresh or conditioned medium (Supplementary Fig. 1c). After an additional 72 h, scaffolds were washed with PBS, and adherent cells were detached by trypsinization. Cells were then resuspended in culture medium to a final volume of 600 μL and analyzed by flow cytometry.

### Semi-quantitative cytokine detection

Semi-quantitative cytokine profiling in culture supernatants was performed using the Human Cytokine Antibody Array C5 (RayBiotech, USA), according to the manufacturer’s instructions. The list of analyzed cytokines is reported in Supplementary Table 1. Conditioned media from 2 and 3D cultures were collected on day 4, kept on ice during collection, and immediately stored at − 80°C to minimize protein degradation. Cell-free medium and BM-MSC conditioned medium were used as controls. After thawing, samples were kept on ice and centrifuged at 10,000 rpm for 10 min at 4°C to remove debris. One milliliter of undiluted supernatant was then applied to each membrane and incubated overnight at 4°C. After washing, membranes were incubated overnight at 4°C with 1 mL of biotinylated antibody cocktail provided in the kit. Following an additional wash step, membranes were incubated with 2 mL HRP-streptavidin for 2 h at room temperature. Signals were developed by incubating membranes for 2 min with 500 μL of detection buffer mixture. Chemiluminescent images were acquired using the ImageQuant LAS 500 system (GE Healthcare, USA). Densitometric analysis was performed with Fiji software according to the manufacturer’s guidelines. All incubation and wash steps were performed on an oscillating rocker to ensure complete membrane coverage.

### Flow cytometry

For apoptosis assays, cells were harvested from 3D cultures by trypsin treatment, resuspended in culture medium, washed with PBS, and stained using an Annexin V assay kit (Immunostep, Spain) according to the manufacturer’s instructions. A total of 10,000 events per sample were acquired. For migration and scaffold colonization assays, the entire 600 μL sample volume was directly analyzed by flow cytometry. Cell number was operationally defined as the number of fluorescent cells acquired during a fixed 5-min acquisition interval. All samples were acquired using a FACSCanto II cytometer (Becton Dickinson, USA), and data were analyzed using FACSDiva software v8.0.2. Once thawed, bone marrow mononuclear cells were stained with PE-Cy7 anti-CD19, APC anti-CD20, and V500 anti-CD45 antibodies (Becton Dickinson, USA) according to the manufacturer’s instructions.

### Immunoblotting

Cells recovered from 3D cultures were lysed in a buffer containing 20 mM Tris, 150 mM NaCl, 2 mM EDTA, 2 mM EGTA, and 0.5% Triton X-100, supplemented with protease inhibitor cocktail (Sigma, USA), 1 mM DTT (Amersham Biosciences, USA), 1 mM PMSF (Sigma, USA), 1 μM okadaic acid (Sigma, USA), and phosphatase inhibitor cocktail (Thermo Fisher Scientific, USA). Protein extracts were separated by SDS-PAGE and transferred onto PVDF membranes (Pierce Biotechnology, USA). Membranes were incubated with primary antibodies against AKT, phospho-AKT (Ser473), S6, phospho-S6 (Ser240/244), GSK3α/β (Santa Cruz Biotechnology, USA), and phospho-GSK3α/β (Ser9/21) (Cell Signaling, USA), followed by the appropriate HRP-conjugated secondary antibodies (Cell Signaling, USA). Immunoreactive bands were visualized using a luminol-based chemiluminescence substrate and acquired with the ImageQuant LAS 500 system. Band densitometry was performed using ImageQuantTL software. Because partial matrix digestion during scaffold processing may affect total protein recovery and housekeeping protein intensity in ECM-derived samples, densitometric values were normalized to GAPDH and interpreted across multiple independent experiments. Quantitative results therefore represent averaged values from replicated analyses rather than a single representative blot.

### Statistical analysis

Data were analyzed using two-tailed unpaired Student’s t test, Wilcoxon matched-pairs signed-rank test, or one-way ANOVA followed by Tukey’s post hoc test, as appropriate. A p value < 0.05 was considered statistically significant. Principal component analysis (PCA) was performed using R software v4.5.0.

## Results

### ECM remodeling reshapes the DLBCL cytokine milieu

**Fig. 1:**
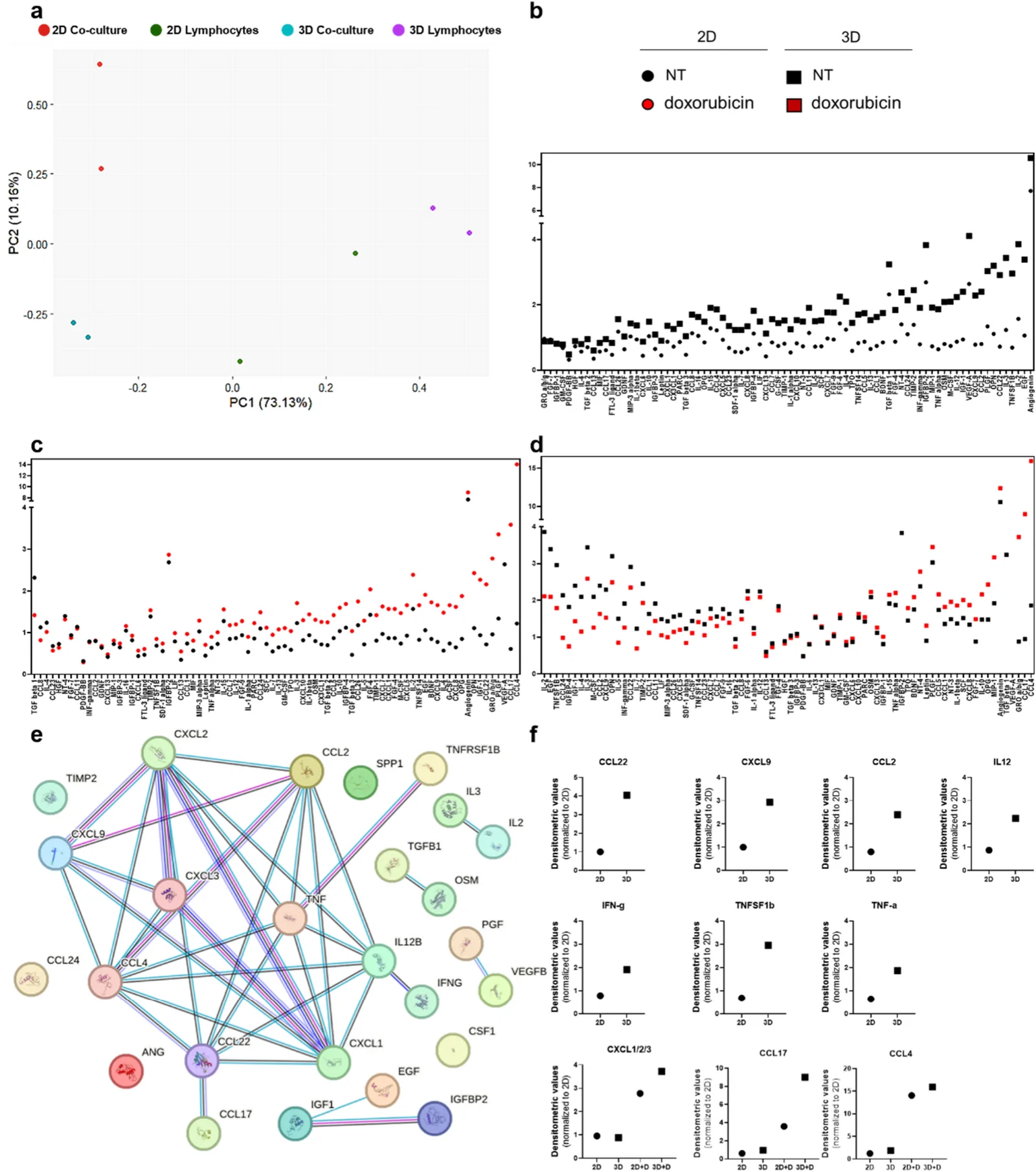
Cytokine profiling of OCI-LY18 in 2D vs 3D culture conditions. (**a**) PCA analysis performed by R 4.5.0 software showing clear separation of cytokine secretion profiles between 2D and 3D cultures. (b–d) Scatter plots comparing relative cytokine abundance: (**b**) untreated 2D vs 3D; (**c**) 2D treated with doxorubicin vs untreated; (**d**) 3D treated with doxorubicin vs untreated. Red symbols indicate doxorubicin-treated cultures. (**e**) STRING protein–protein interaction network of cytokines upregulated in 3D conditions, highlighting a highly interconnected chemokine cluster (26 nodes, 37 edges; clustering coefficient 0.698; p < 1×10⁻^1^⁶). (**f**) Representative cytokines most affected by 3D culture, plotted as relative abundance compared with 2D untreated controls

To investigate the impact of ECM architecture on cytokine secretion, we profiled 80 cytokines in OCI-LY18 supernatants cultured under 2D or 3D conditions, in the presence or absence of doxorubicin (Fig. 1). Cell proliferation was comparable across systems, validating the analysis. (Supplementary Fig. 3). Principal component analysis (PCA) clearly separated 3D from 2D cultures, indicating a major effect of ECM dimensionality on the cytokine milieu (Fig. 1a). Under basal conditions, 24 cytokines were upregulated more than twofold in 3D cultures, including CCL2, CCL22, VEGF, TNFRSF1, CXCL9, IL-2, IL-3, and angiogenin (Fig. 1b; Supplementary Fig. 5). In contrast, cytokine changes induced by doxorubicin in 2D cultures were attenuated or lost in 3D, suggesting that ECM-derived signals override chemotherapy-driven modulation (Fig. 1c–d; Supplementary Fig. 5).

STRING network analysis of cytokines upregulated in 3D revealed a highly interconnected network (26 nodes, 37 edges; clustering coefficient 0.698; p < 1 × 10⁻1⁶) (Fig. 1e). Gene Ontology analysis confirmed enrichment in leukocyte migration and chemokine-mediated signaling pathways. Relative cytokine levels are shown in Fig. 1f, supporting the emergence of a coordinated pro-migratory secretome induced by ECM engagement.

### ECM-conditioned media enhance DLBCL migration

**Fig. 2:**
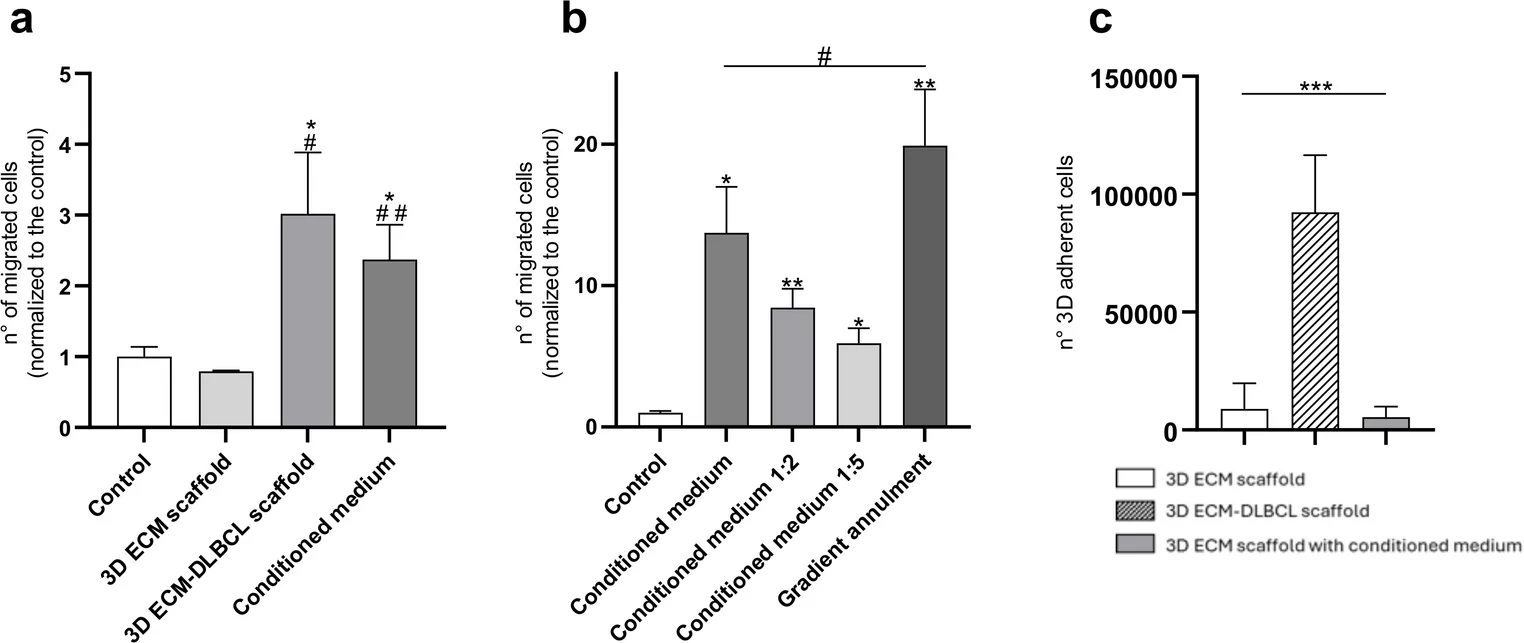
ECM-derived cytokines promote migration and scaffold colonization of OCI-LY18 cells. (**a**) Transwell migration assay showing enhanced migration toward conditioned medium and 3D ECM models compared to scaffold or medium alone (n=6). (**b**) Migration increased with conditioned medium concentration and was maximal when the cytokine gradient was annulled (n=3). Data are mean ± SD; *p<0.05, **p<0.01. (**c**) Scaffold colonization was significantly higher when scaffolds were pre-seeded with DLBCL cells (Statistical analysis: ONE-way ANOVA ***=p<0.0005, n=3). (**c**)

To assess the functional relevance of ECM-induced cytokine remodeling, we evaluated the chemotactic activity of conditioned media derived from 3D cultures. Transwell migration assays showed a marked increase in OCI-LY18 migration compared with fresh medium or decellularized scaffold controls (≈threefold; *with respect to the control conditions p < 0.05. #with respect to scaffold conditions. #p < 0.05; ##p < 0.005. Figure 2a). Dilution of conditioned medium progressively reduced migration, indicating a concentration-dependent effect (Fig. 2b). Unexpectedly, when conditioned medium was added to both chambers, thereby abolishing the chemokine gradient, migration further increased (One-way ANOVA test: #p < 0.05; Fig. 2c). These findings suggest that DLBCL migration is primarily driven by absolute cytokine concentration rather than by directional gradients.

### ECM-adherent cells promote secondary scaffold colonization

We next investigated whether ECM-adherent DLBCL cells could promote further recruitment of tumor cells. SYTO-labeled OCI-LY18 cells seeded onto pre-colonized scaffolds showed a > tenfold increase in recruitment compared with decellularized scaffolds or scaffolds exposed only to conditioned medium (ECM scaffold: 8,957 ± 10,867 cells; ECM-DLBCL scaffold: 92,268 ± 24,263 cells; ECM + conditioned medium: 5,468 ± 4,442 cells; p < 0.0005, n = 3). Figure 2c) These results indicate that ECM–tumor interactions generate a positive feedback loop that enhances DLBCL recruitment and scaffold colonization.

### ECM adhesion reduces sensitivity to ibrutinib

**Fig. 3:**
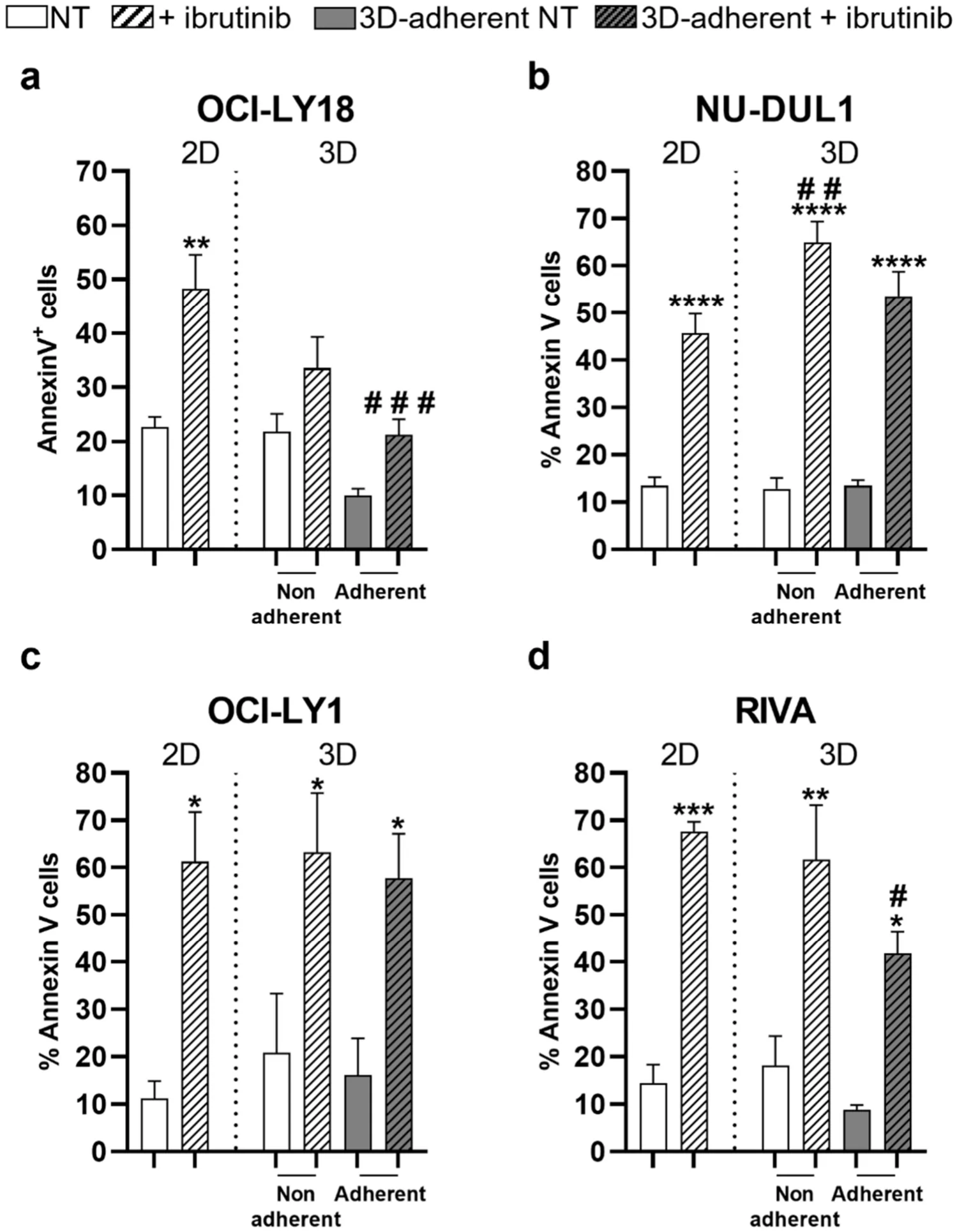
Annexin V cytofluorimetric analysis of DLBCL cell lines after ibrutinib treatment. Ibrutinib treatment was effective in apoptosis induction in all four tested cell 2D lines. **a, d**: the apoptotic effect of ibrutinib is significantly reduced in 3D-adherent OCI-LY18 and RIVA cell vs 2D controls (n=3–7). Statistical analysis: ONE-way ANOVA and Tukey’s post hoc test * with respect to the non-treated controls: *= p<0.05; **= p<0.005; ***= p<0.0005; ****= p<0.0001. # with respect to the 2D treated controls: #= p<0.05; ##= p<0.01; ### p<0.005). Error bars represent standard error mean

We then evaluated whether ECM adhesion modulates sensitivity to ibrutinib. In 2D conditions, all four DLBCL cell lines showed significant apoptosis following ibrutinib treatment. In contrast, 3D-adherent OCI-LY18 and RIVA cells displayed markedly reduced sensitivity (21.2 ± 5.5% vs. 48.3 ± 13.9% Annexin V positive cells for OCI-LY18; 41.8 ± 8.1% vs. 67.6 ± 3.6% Annexin V positive cells for RIVA) (* with respect to the non-treated controls: * = p < 0.05; ** = p < 0.005; *** = p < 0.0005; **** = p < 0.0001. # With respect to the 2D treated controls: # = p < 0.05; ## = p < 0.01; ### p < 0.005; Fig. 3). No difference between 2 and 3D conditions was observed for OCI-LY1 and NU-DUL-1. The inclusion of BM-MSCs resulted in modest and variable trends toward reduced apoptosis, but these did not reach statistical significance. In contrast, reduced sensitivity was consistently observed only in ECM-adherent OCI-LY18 and RIVA cells, indicating that direct ECM–tumor interaction is the dominant driver of this effect (Supplementary Fig. 4).

### ECM-induced drug resistance is reversible and adhesion-dependent

To determine whether ECM-induced resistance was stable or dependent on adhesion, OCI-LY18 cells previously cultured in 3D (primed cells) were transferred back to 2D conditions.

**Fig. 4:**
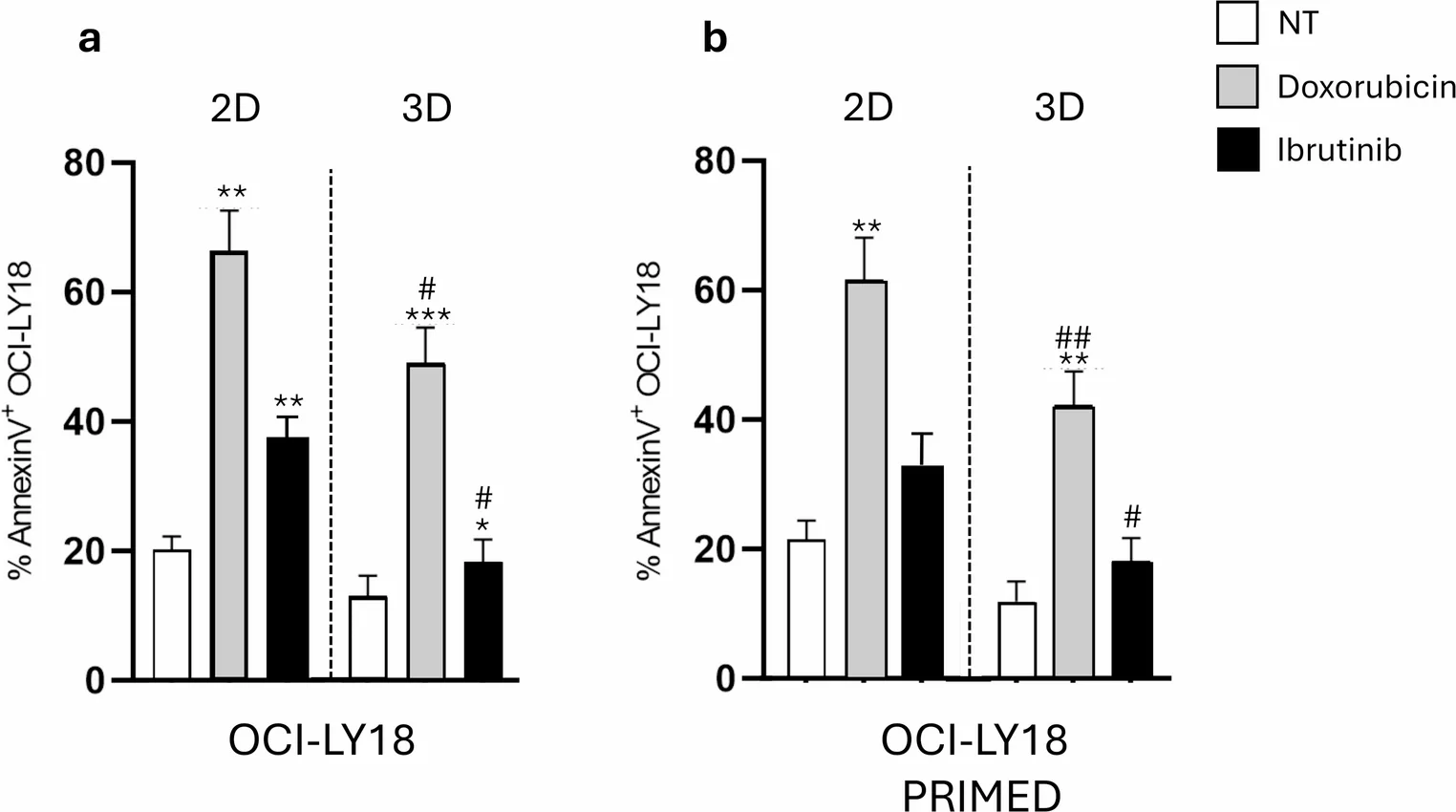
Annexin V cytofluorimetric analysis of primed OCI-LY18 readapted to 2D from a previous 3D culture. Cells were cultured for 72h in adhesion to 3D ECM scaffold, then harvested and expanded in a 2D canonical culture. Drug sensitivity was restored when primed (**b**) cells were treated in a 2D conditions showing no significant differences with normal OCI-LY18 (**a**). Within each culture conditions (2D or 3D), differences among untreated, doxorubicin-treated, and ibrutinib-treated cells were assessed using one-way repeated-measures ANOVA and Tukey’s post hoc test. Direct comparisons between 2D naïve cells (panel A) and 2D primed/readapted cells (panel B) were performed using paired t-tests, as these conditions represent matched measurements from the same independent experiments* with respect to the non-treated controls: *p<0.05; **p<0.01; ***p<0.001. # with respect to the 2D treated controls: #= p<0.05, ##=p<0.01; n=9

Primed cells retained reduced sensitivity to both doxorubicin and ibrutinib when maintained in 3D. However, upon transfer to 2D conditions, drug sensitivity was restored (doxorubicin: 60.4 ± 15.6% vs. 66.3 ± 18.6%; ibrutinib: 34.4 ± 7.1% vs 38.1 ± 10.1%; n = 9) (*p < 0.05; **p < 0.01; ***p < 0.001; # with respect to the 2D treated controls: # = p < 0.05, ## = p < 0.01; Fig. 4). These findings indicate that ECM-mediated resistance is reversible and strictly dependent on ongoing cell–ECM interaction.

### ECM-specific signals, rather than 3D architecture, drive drug resistance

**Fig. 5.**
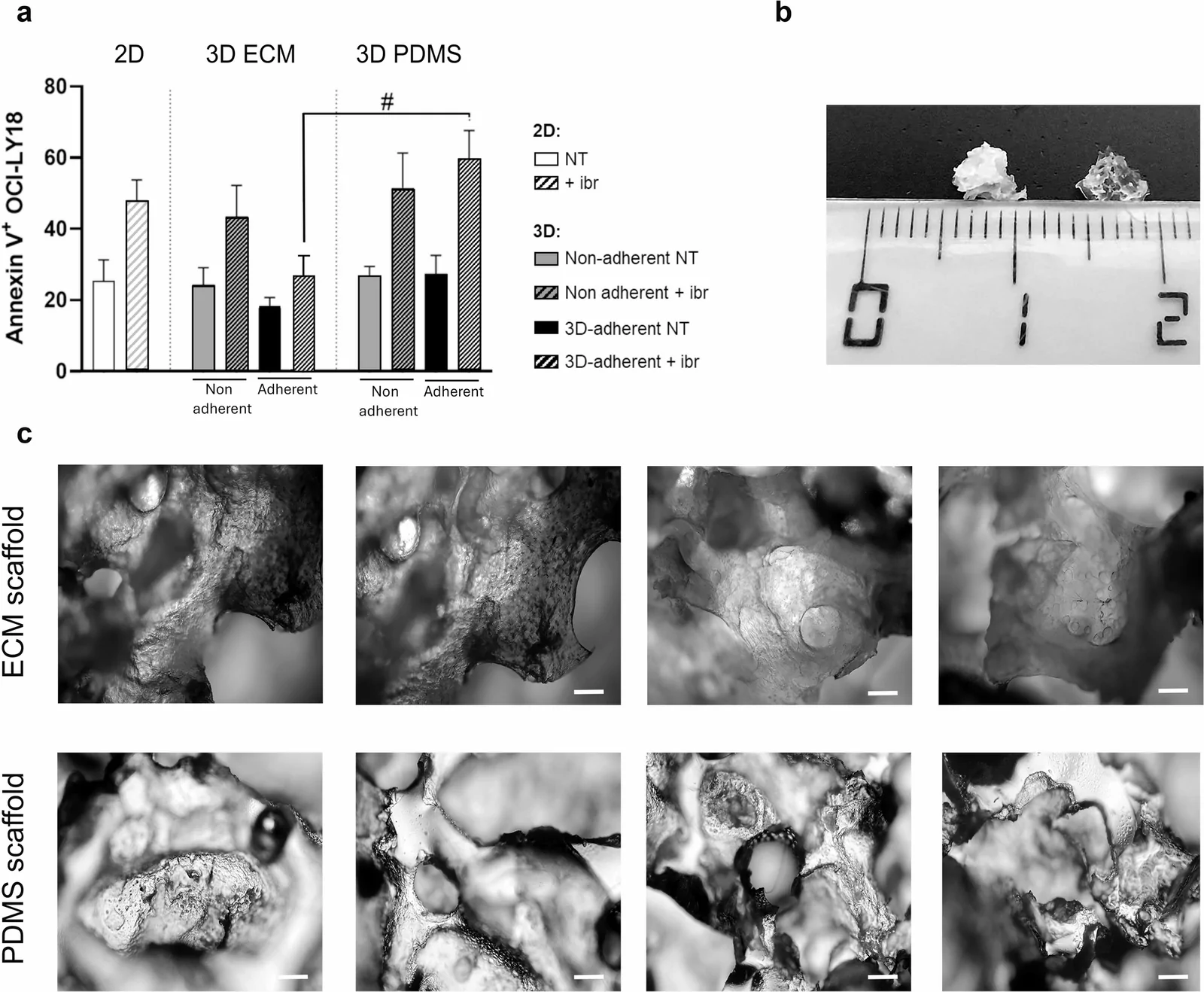
**a**) Annexin V cytofluorimetric analysis of OCI-LY18 cells cultured in standard 2D conditions, on bone-derived 3D ECM scaffolds, or on inert 3D PDMS scaffolds and treated with ibrutinib. In 3D models, adherent and non-adherent fractions were analyzed separately. The reduction of ibrutinib-induced apoptosis determined by the 3D ECM scaffold is not visible in 3D PDMS cultures (one-way ANOVA followed by Tukey’s post hoc test). #p < 0.05. Error bars represent SEM. *n* = 3 independent experiments. **b**) comparison between ECM- and PDMS-based 3D scaffolds. **c**) Optical microscope images of ECM- and PDMS-based 3D scaffolds. No substantial differences were noticed between the scaffolds. Scale bars: 400 µm

To distinguish between structural and biochemical effects, we compared bone-derived ECM scaffolds with inert PDMS scaffolds. Baseline apoptosis of OCI-LY18 cells was comparable across PDMS, ECM, and 2D conditions (27.4 ± 8.9%, 17.8 ± 5.1%, and 25.4 ± 10.1% Annexin V^+^ OCI-LY18) (Fig. 5a). However, following ibrutinib treatment, PDMS-adherent cells remained fully sensitive and displayed significantly higher apoptosis compared with ECM-adherent cells (PDMS: 59.8 ± 13.6%; ECM: 27.2 ± 8.5%; 2D: 48.1 ± 9.9%; n = 3) (#p < 0.05; Fig. 5a). Despite similar macroscopic structure and pore organization (Fig. 5b and c), only ECM scaffolds conferred drug resistance, indicating that biochemical ECM-specific signals, rather than 3D architecture alone, mediate this effect.

### ECM adhesion activates the AKT/mTOR pathway

**Fig. 6:**
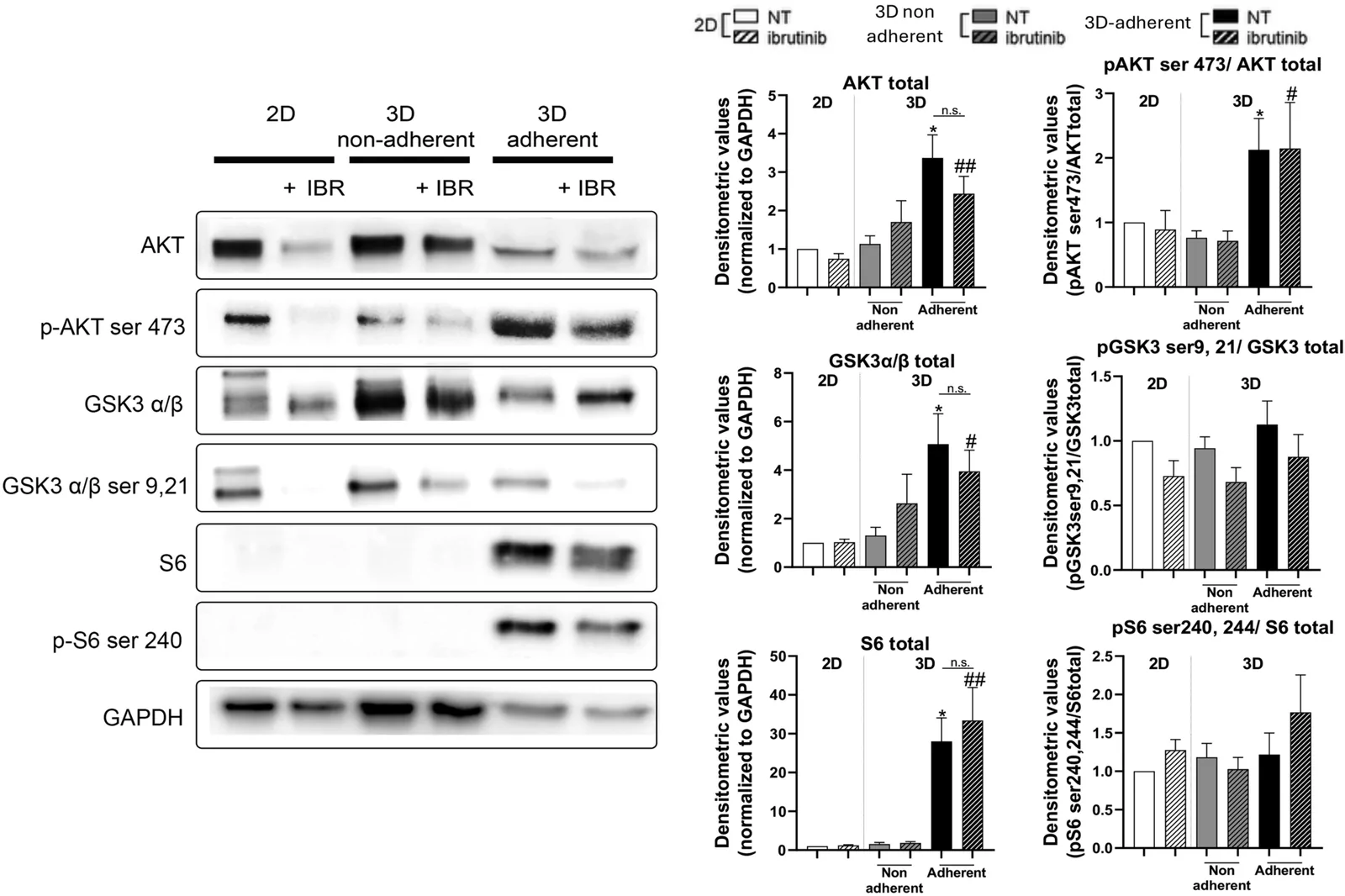
Western blot analysis of AKT, pAKT (ser473), S6, pS6 (ser 240/244), GSK3 α/β, and pGSK3 α/β (ser 9/21) in OCI-LY18 cells cultured under different conditions. The six experimental groups include: 2D untreated cells, 2D ibrutinib-treated cells, 3D ECM-adherent untreated cells, 3D ECM-adherent ibrutinib-treated cells, 3D non-adherent untreated cells, and 3D non-adherent ibrutinib-treated cells. Densitometric analyses were performed using ImageQuant TL software and normalized as indicated. Statistical analysis: *non-parametric Wilcoxon matched-pairs signed-rank test* with respect to the 2D untreated conditions (*p < 0.05; # *parametric t-test* with respect to the 2D treated conditions (#p < 0.05). Error bars represent standard error of the mean; n = 7. Variability in GAPDH signal intensity across lanes reflects differences in protein recovery from 3D ECM-derived samples; densitometric quantification is based on normalization to GAPDH across multiple independent experiments

Given the known role of the PI3K/AKT/mTOR pathway in resistance to BTK inhibition, we analyzed key signaling components in OCI-LY18 cells [8, 9]. Specifically, we assessed total AKT, phosphorylated AKT (Ser473), S6, phosphorylated S6 (Ser 240/244), GSK3 α/β, and phosphorylated GSK3 α/β (Ser 9/21) by Western blot analysis (Fig. 6). 3D-adherent cells showed increased total AKT expression (3.4 ± 1.6-fold vs 2D, p < 0.05. n = 7) and increased AKT phosphorylation (2.1 ± 1.6-fold) (*p < 0.05; # parametric t-test with respect to the 2D treated conditions; #p < 0.05; Fig. 6). GSK3 α/β expression was also elevated in 3D adherent cells compared to 2D cultures (5.1 ± 3.3-fold; p < 0.05), while phosphorylation remained unchanged (Fig. 6). S6 protein levels were markedly increased in 3D-adherent cells under both untreated and ibrutinib-treated conditions (28.1 ± 15.9-fold and 33.4 ± 22.6-fold, respectively; p < 0.05), whereas pS6 levels did not significantly change (Fig. 6). Importantly, these differences were driven by ECM adhesion rather than drug exposure, indicating that ECM engagement promotes a constitutive pro-survival signaling state independent of ibrutinib treatment.

## Discussion

The bone marrow microenvironment is increasingly recognized as a key regulator of lymphoma biology and treatment response. In this study, a human bone-derived ECM scaffold reproduced critical tumor–matrix interactions and revealed their impact on cytokine secretion and drug sensitivity. These findings support and extend the concept of cell adhesion–mediated drug resistance (CAM-DR), previously described in myeloma and leukemia, to DLBCL, where direct ECM engagement reshapes tumor cell behavior [10, 11].

A central observation of our study is that ibrutinib tolerance is not uniform but emerges selectively in specific DLBCL cell lines and is reversible upon disruption of cell–ECM interaction. This indicates that resistance is not a fixed trait but a context-dependent and dynamically regulated state. Similar mechanisms have been reported in chronic lymphocytic leukemia, where stromal interactions confer transient protection from BTK inhibition [12]. Notably, BM-MSCs did not significantly affect drug response, suggesting that direct ECM–tumor interaction, rather than stromal support, is the dominant contributor to reduced ibrutinib sensitivity. Consistently, inert PDMS scaffolds failed to reproduce this effect, indicating that biochemical ECM-derived signals, rather than 3D structure alone, are required.

The heterogeneity of ECM-mediated modulation across DLBCL cell lines likely reflects differences in intrinsic signaling dependencies and microenvironmental responsiveness. Variability in BCR signaling reliance, baseline activation of survival pathways, or differential expression of adhesion molecules and integrins may influence how individual cell lines integrate ECM-derived cues. Although a systematic dissection of these mechanisms was beyond the scope of this study, our findings underscore that microenvironment-driven drug modulation cannot be predicted solely by cell-of-origin classification [13–15].

At the mechanistic level, ECM adhesion was associated with activation of the AKT/mTOR axis, as evidenced by increased AKT and S6 expression. This pathway is a well-established mediator of resistance to BTK inhibition and a hallmark of aggressive DLBCL biology [16, 17]. The marked upregulation of S6, in particular, is notable given its association with high-risk lymphoma phenotypes, including MYC/BCL2 double-expressor disease and Richter transformation [17]. While our data are correlative, they support a model in which ECM-driven activation of AKT/mTOR provides a survival pathway independent of BCR/BTK signaling. This is consistent with preclinical evidence showing that combined targeting of BTK and PI3K/mTOR pathways can overcome resistance [18].

In addition to drug resistance, ECM interaction induced a coordinated remodeling of the cytokine milieu. The observed increase in chemokines such as CCL2, CCL22, and CXCL9 defines a functionally interconnected, pro-migratory network that enhances tumor cell recruitment and scaffold colonization. These findings are consistent with previous evidence that ECM components shape inflammatory signaling and facilitate tumor dissemination within the bone marrow niche [19–22]. Such a positive feedback loop between ECM adhesion, cytokine secretion, and enhanced tumor recruitment may help explain the adverse prognostic impact of bone marrow infiltration in DLBCL patients [23]. Importantly, our data support a model of collective cytokine activity rather than a single dominant mediator. Functional studies will be required to dissect the contribution of individual cytokines.

These results have several translational implications. First, they highlight the limitations of conventional 2D and spheroid models in capturing ECM-dependent mechanisms of drug resistance. Second, they provide a rationale for combinatorial strategies targeting both BTK and downstream survival pathways, such as AKT/mTOR or integrin-mediated signaling. Third, they emphasize the dynamic and reversible nature of microenvironment-driven resistance, suggesting that therapeutic sequencing or microenvironment-targeting approaches may enhance treatment efficacy.

An important consideration is that the concentrations of ibrutinib required to induce apoptosis in vitro exceed those achieved in patients. This is a common feature of 3D and ex vivo systems, where limited drug penetration and microenvironmental protection necessitate higher nominal concentrations to elicit measurable biological effects. Accordingly, our experimental design was not intended to model pharmacokinetics but to compare relative drug sensitivity across different microenvironmental contexts.

This study has several limitations. All experiments were performed in established DLBCL cell lines, which do not fully capture the genetic and microenvironmental heterogeneity of primary tumors. In addition, although the decellularized scaffold preserves key ECM features, it lacks the cellular complexity of the bone marrow niche. The model was intentionally designed to isolate ECM-specific effects; however, future studies should integrate stromal, endothelial, and immune components to better recapitulate in vivo conditions [24, 25]. Finally, the association between ECM adhesion and AKT/mTOR activation remains correlative, and functional validation will be required to establish causality.

In conclusion, the bone-derived 3D model provides a physiologically relevant platform to dissect ECM–DLBCL interactions. By linking ECM adhesion to reversible drug resistance, cytokine remodeling, and activation of pro-survival signaling pathways, our study defines a framework for understanding microenvironment-driven therapy resistance and for developing more effective therapeutic strategies in bone marrow–infiltrating DLBCL.

## Supplementary Information

Below is the link to the electronic supplementary material.

Supplementary file1.

## Data Availability

The data that support the findings of this study are available on request from the corresponding author.
