# Correction to: DLBCL-microenvironment interactions: cytokine profiling and ECM mediated ibrutinib resistance in a 3D bone-based model

**DOI:** 10.1007/s12032-026-03356-w

**Published:** 2026-08-26

**Authors:** Jessica Ceccato, Maria Piazza, Giulia Gualtiero, Samuela Carraro, Francesco Cinetto, Carlo Biz, Sabrina Manni, Marco Pizzi, Sabrina Pianalto, Simone Zoletto, Valeria Carabotta, Nicolo’ Danesin, Pietro Ruggieri, Angelo Paolo Dei Tos, Francesco Piazza, Livio Trentin, Fabrizio Vianello

**Affiliations:** 1 Hematology Unit, Department of Medicine–DIMED, University-Hospital of Padua, Padua, Italy; 2 Laboratory of Myeloma and Lymphoma Pathobiology, Veneto Institute of Molecular Medicine (VIMM), Padua, Italy; 3 Internal Medicine and Allergology and Clinical Immunology Unit, Treviso Ca’ Foncello Hospital, Treviso, Italy; 4 Orthopedics and Orthopedic Oncology, Department of Surgery, Oncology and Gastroenterology (DiSCOG), University-Hospital of Padua, Padua, Italy; 5 Pathology Unit, Department of Medicine-DIMED, University-Hospital of Padua, Padua, Italy

**Correction to: Medical Oncology**

**https://doi.org/10.1007/s12032-026-03309-3**

Following publication of the article, the authors identified areas of Figures 2, 4 and 5 which were inconsistent with the rest of the article’s figures. To improve readability and the reader’s ease of interpretation, minor adjustments have been made to the figures, as outlined below.

Figure 2: Panel C has been resized and aligned with panels A and B.

Figure 4: The missing y-axis label has been added to panel B; “Not treated” has been replaced with “NT” for consistency with all other figures ; the dashed lines have been changed from blue to black.

Figure 5: The letters “a” and “b” have been aligned; the text “3D ECM” has been placed on a single line; the labels and bars below the graph have been aligned; “Scafold” has been corrected to “Scaffold.”; the letters and text have been changed from grey to black.

For completeness and transparency, the original (incorrect) and corrected versions of Figures 2 and 4, and 5 are displayed below.

Incorrect Figure 2:

**Fig. 2:**
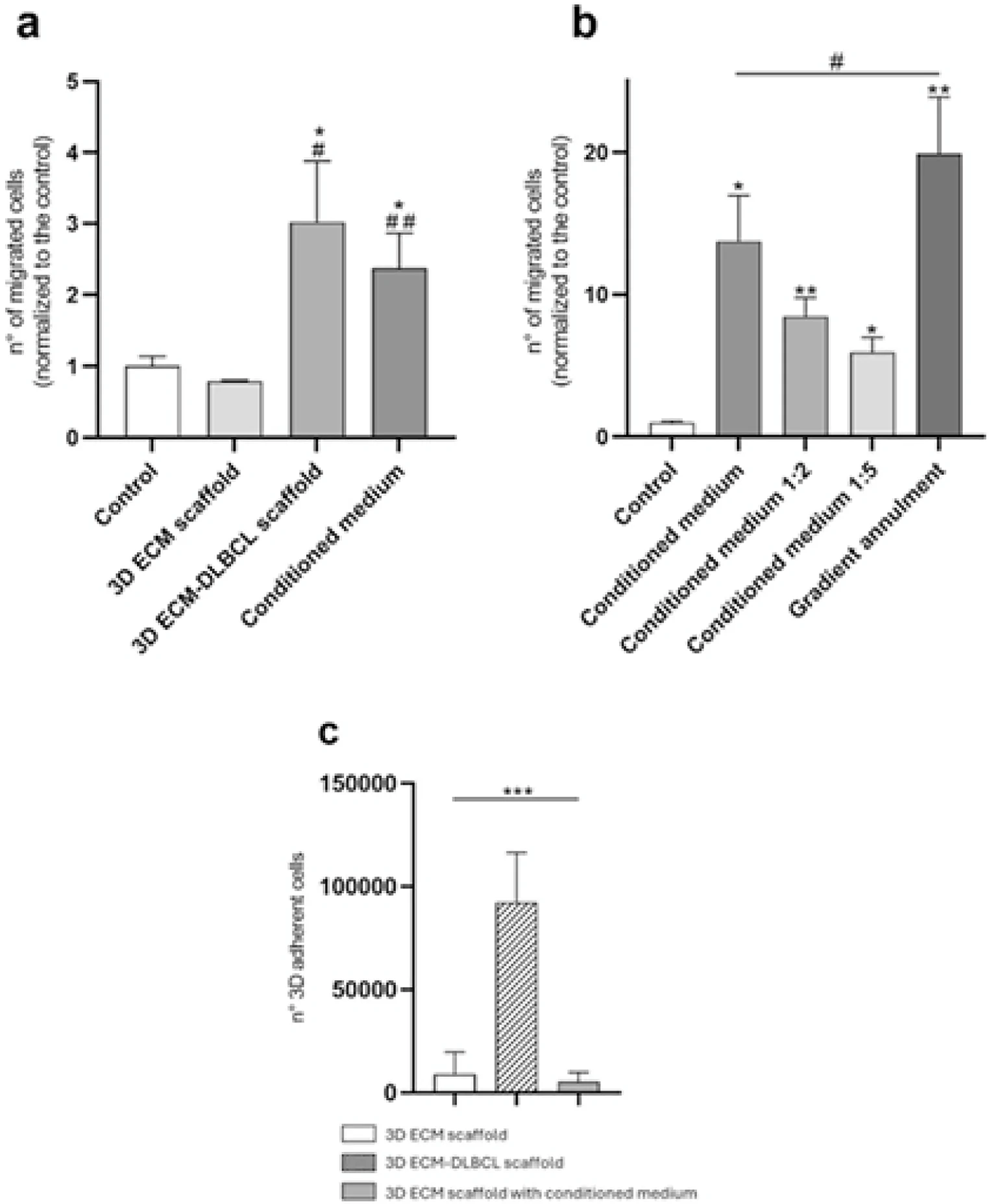
ECM-derived cytokines promote migration and scaffold colonization of OCI-LY18 cells. (**a**) Transwell migration assay showing enhanced migration toward conditioned medium and 3D ECM odels compared to scaffold or medium alone (*n* = 6). (**b**) Migration increased with conditioned medium oncentration and was maximal when the cytokine gradient was annulled (*n* = 3). Data are mean ± SD; *p < 0.05, **p < 0.01. (c) Scaffold colonization was significantly higher when scaffolds were pre-seeded with DLBCL cells (Statistical analysis: ONE-way ANOVA ***=*p* < 0.0005, *n* = 3). (**c**)

Corrected Figure 2:

**Fig. 2:**
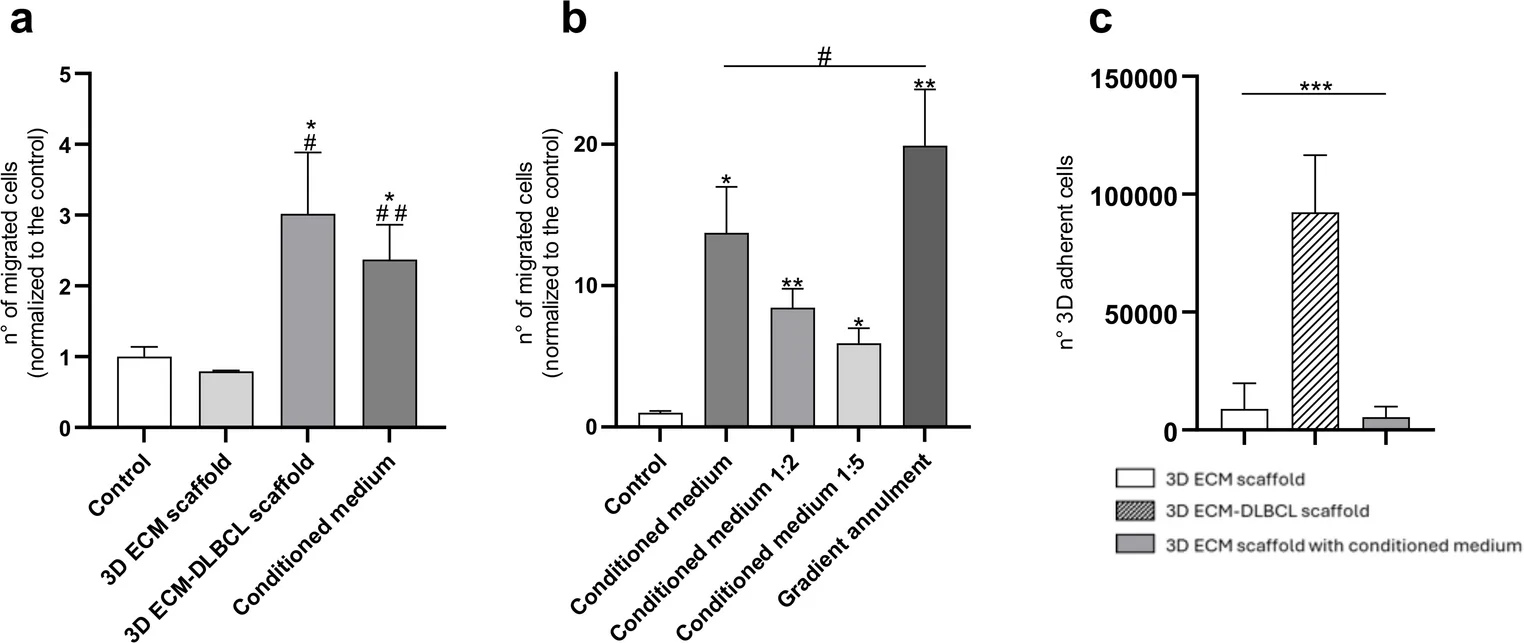
ECM-derived cytokines promote migration and scaffold colonization of OCI-LY18 cells. (**a**) Transwell migration assay showing enhanced migration toward conditioned medium and 3D ECM odels compared to scaffold or medium alone (*n* = 6). (**b**) Migration increased with conditioned medium oncentration and was maximal when the cytokine gradient was annulled (*n* = 3). Data are mean ± SD; *p < 0.05, **p < 0.01. (c) Scaffold colonization was significantly higher when scaffolds were pre-seeded with DLBCL cells (Statistical analysis: ONE-way ANOVA ***=*p* < 0.0005, *n* = 3). (**c**)

Incorrect Figure 4:

**Fig. 4:**
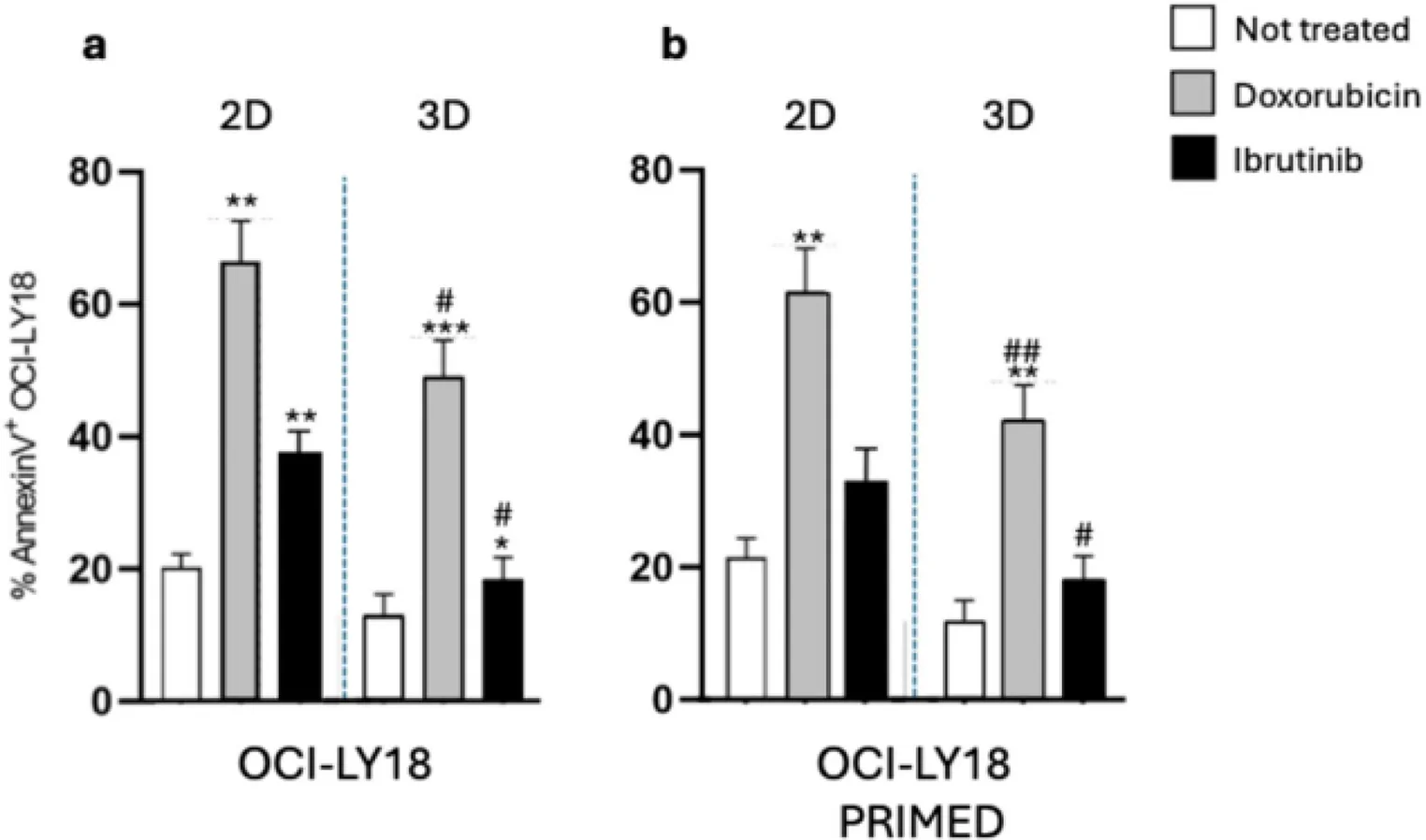
Annexin V cytofluorimetric analysis of primed OCI-LY18 readapted to 2D from a previous 3D culture. Cells were cultured for 72 h in adhesion to 3D ECM scaffold, then harvested and expanded in a 2D canonical culture. Drug sensitivity was restored when primed (**b**) cells were treated in a 2D conditions showing no significant differences with normal OCI-LY18 (**a**). Within each culture conditions (2D or 3D), differences among untreated, doxorubicin-treated, and ibrutinib-treated cells were assessed using one-way repeated-measures ANOVA and Tukey’s post hoc test. Direct comparisons between 2D naïve cells (panel A) and 2D primed/readapted cells (panel B) were performed using paired t-tests, as these conditions represent matched measurements from the same independent experiments. * with respect to the non-treated controls: *p < 0.05; **p < 0.01; ***p < 0.001. # with respect to the 2D treated controls: #= *p* < 0.05, ##=*p* < 0.01; *n* = 9

Corrected Figure 4:

**Fig. 4:**
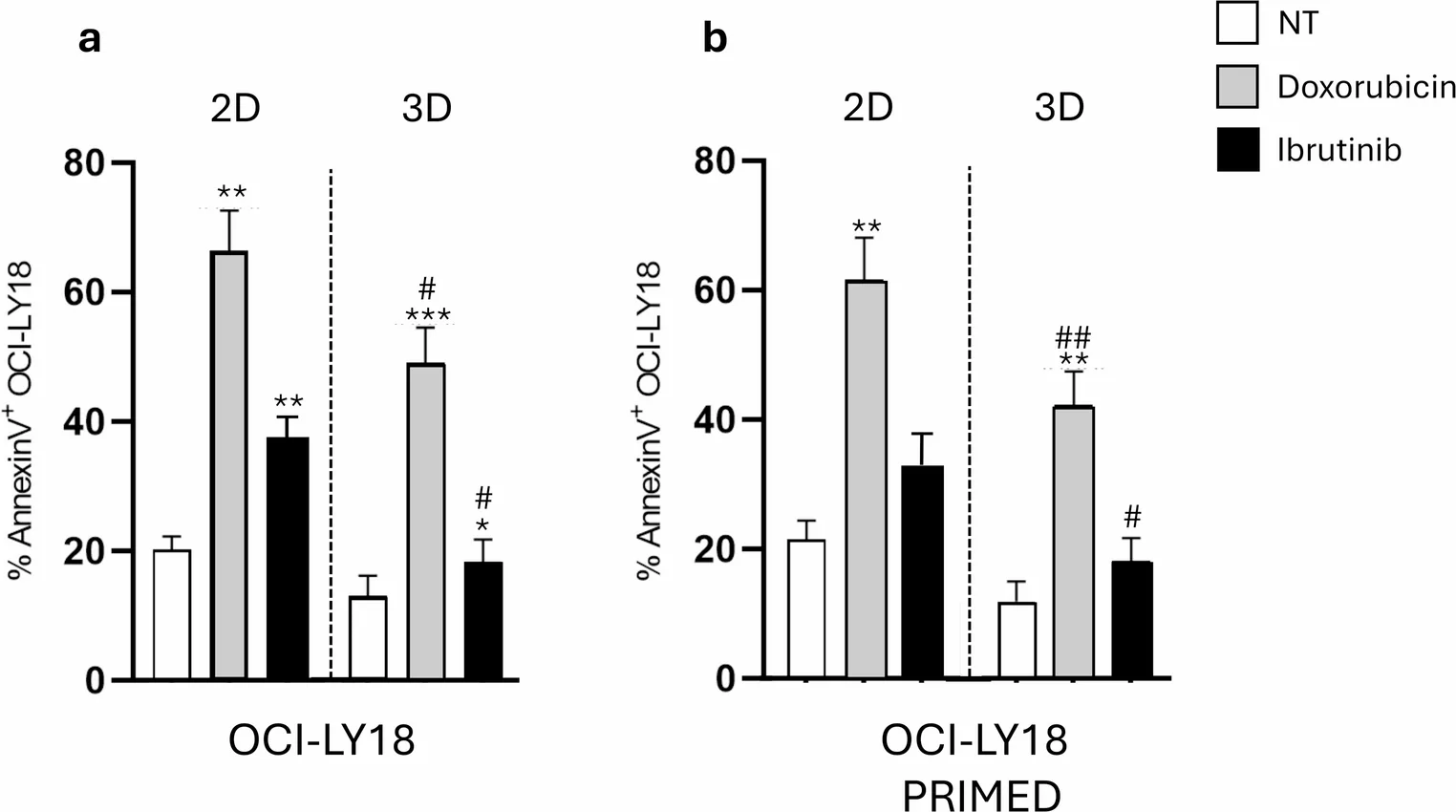
Annexin V cytofluorimetric analysis of primed OCI-LY18 readapted to 2D from a previous 3D culture. Cells were cultured for 72 h in adhesion to 3D ECM scaffold, then harvested and expanded in a 2D canonical culture. Drug sensitivity was restored when primed (**b**) cells were treated in a 2D conditions showing no significant differences with normal OCI-LY18 (**a**). Within each culture conditions (2D or 3D), differences among untreated, doxorubicin-treated, and ibrutinib-treated cells were assessed using one-way repeated-measures ANOVA and Tukey’s post hoc test. Direct comparisons between 2D naïve cells (panel A) and 2D primed/readapted cells (panel B) were performed using paired t-tests, as these conditions represent matched measurements from the same independent experiments. * with respect to the non-treated controls: *p < 0.05; **p < 0.01; ***p < 0.001. # with respect to the 2D treated controls: #= *p* < 0.05, ##=*p* < 0.01; *n* = 9.

Incorrect Figure 5:

**Fig. 5:**
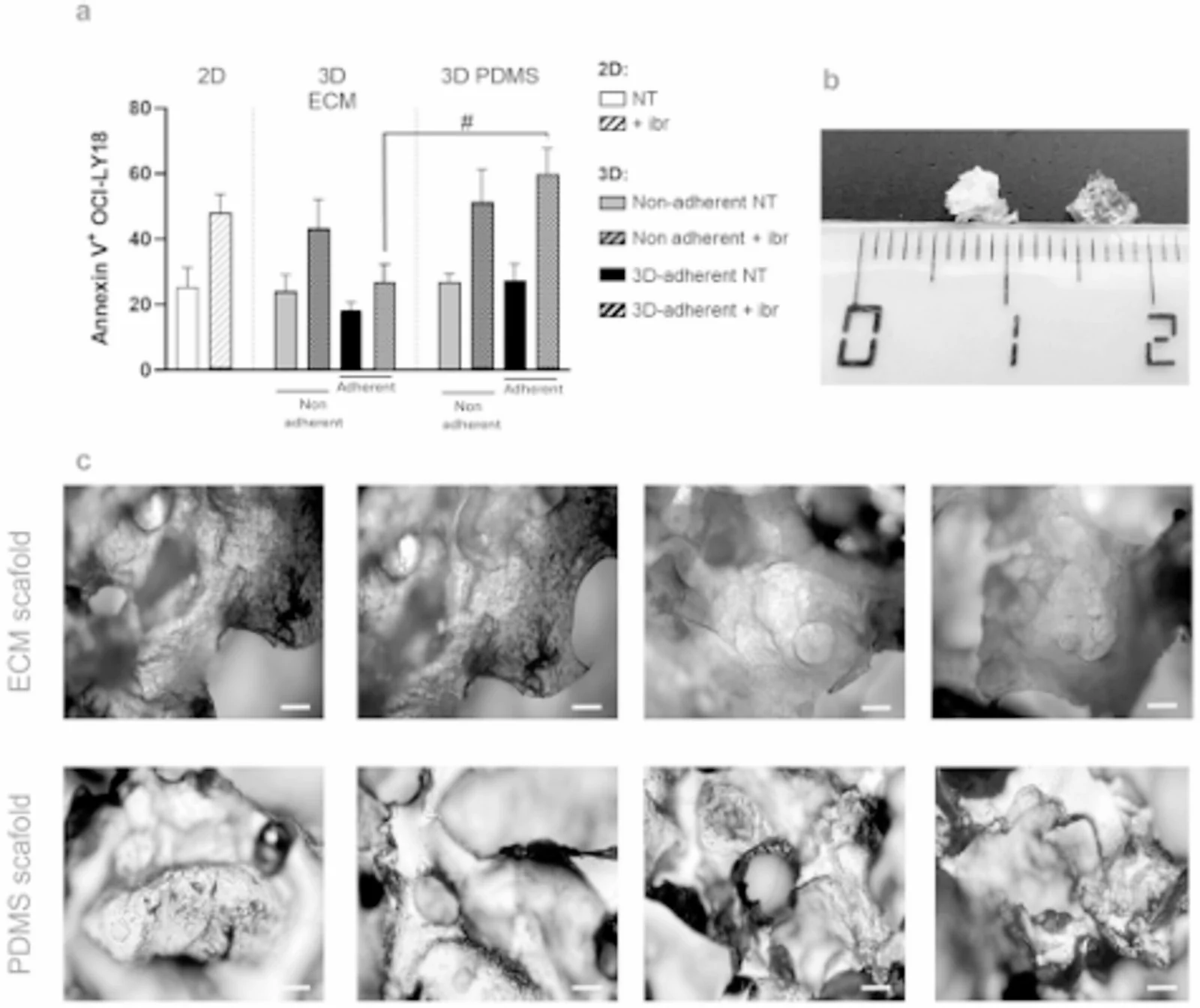
**(a)** Annexin V cytofluorimetric analysis of OCI-LY18 cells cultured in standard 2D conditions, on bone-derived 3D ECM scaffolds, or on inert 3D PDMS scaffolds and treated with ibrutinib. In 3D models, adherent and non-adherent fractions were analyzed separately. The reduction of ibrutinib-induced apoptosis determined by the 3D ECM scaffold is not visible in 3D PDMS cultures (one-way ANOVA followed by Tukey’s post hoc test). #*p* < 0.05. Error bars represent SEM. *n* = 3 independent experiments. **(b)** comparison between ECM- and PDMS-based 3D scaffolds. **(c)** Optical microscope images of ECM- and PDMS-based 3D scaffolds. No substantial differences were noticed between the scaffolds. Scale bars: 400 μm

Corrected Figure 5:

**Fig. 5:**
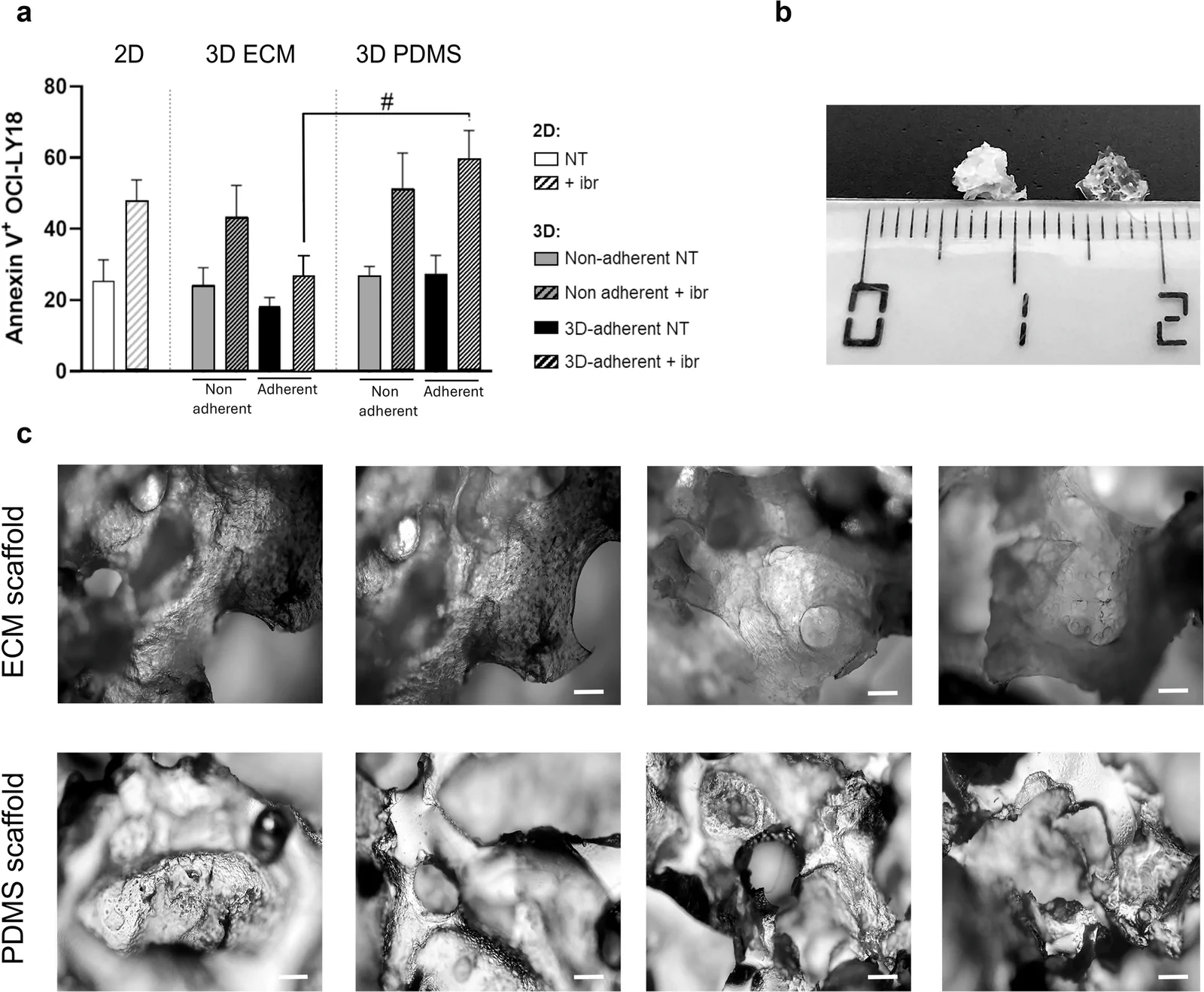
**(a)** Annexin V cytofluorimetric analysis of OCI-LY18 cells cultured in standard 2D conditions, on bone-derived 3D ECM scaffolds, or on inert 3D PDMS scaffolds and treated with ibrutinib. In 3D models, adherent and non-adherent fractions were analyzed separately. The reduction of ibrutinib-induced apoptosis determined by the 3D ECM scaffold is not visible in 3D PDMS cultures (one-way ANOVA followed by Tukey’s post hoc test). #*p* < 0.05. Error bars represent SEM. *n* = 3 independent experiments. **(b)** comparison between ECM- and PDMS-based 3D scaffolds. **(c)** Optical microscope images of ECM- and PDMS-based 3D scaffolds. No substantial differences were noticed between the scaffolds. Scale bars: 400 μm.

The original article has been corrected.

