# Supplementary material for: DLBCL-microenvironment interactions: cytokine profiling and ECM-mediated ibrutinib resistance in a 3D bone-based model
Source: Med Oncol. 2026 Jul 15;43(9):220. doi: 10.1007/s12032-026-03309-3 (PMC13372843; doi:10.1007/s12032-026-03309-3)

**Supplementary Methods**

**CFSE proliferation assay**

Carboxyfluorescein succinimidyl ester (CFSE), was added at the final concentration of 10 μM to 0.5 × 10^6^ OCI-LY18 cells according to manufacturer’s recommendations. The CFSE testing concentration was established by dose response curve analysis (data not shown). CFSE-stained cells were cultured in the presence of 3D ECM scaffold and the relative fluorescent signals were then determined via cytofluorimetric analysis at days 1, 2, 3, and 4.

**Bone Marrow Mesenchymal Stromal Cells (BM-MSCs) isolation**

For Bone Marrow Mesenchymal Stromal Cells (BM-MSCs) isolation, 3 ml of BM aspirate were obtained from subjects who underwent staging procedures for NHL lymphomas. Only samples that did not show lymphoma infiltration were considered for MSCs cultures. All samples were obtained with written informed consent in accordance with local ethical committee requirements. BM aspirates were processed by red blood cell lysis, washed, and cultured in DMEM with 20% FBS, 1% P/S, 0.01 mg/ml of fungizone, and 0.25 μg/ml of ciprofloxacin, and plated in T25 flasks (Costar, Cambridge, MA, USA). When 90%–95% confluence was reached (in about 30 days), adherent cells were trypsinized (Gibco, UK) and expanded for 3–5 weeks. BM-MSCs were checked for positivity of CD105, CD73, and CD90 and for the lack of expression of CD45 and CD20 (data not shown). BM-MSCs scaffold recellularization was performed by directly seeding 6 × 10^4^ BM-MSCs over the 3D scaffold. For drug treatments, the 3D models were prepared as described above, the same number of BM-MSCs was plated in a 24-well plate to perform the 2D co-culture as previously described (8) and cultured for 4 days.

**Primed cell lines establishment**

OCI-LY18 3D adherent cells, grown onto three 3D ECM-DLBCL models, were harvested by trypsinization and cultured in a 48 well plate with 0.5 ml of growing medium for 2 days and then expanded by splitting the cell suspension 1:2 in two clean wells (48 well plate). 2 days after, cells were pooled together and expanded in 4 wells (250 µl of cell suspension and 250 µl of fresh medium per well). At day 7 cells were pooled together as before and supplemented with fresh growing medium to a final volume of 4 ml and then moved to a 6 well plate. At day 9 cells were split 1:4 in a 6 well plate and let in culture for further 2 days. After that, cells were collected, resuspended in 8 ml of PBS and stratified over 4 ml of Lymphocyte Separation Media (Lymphosep. Biowest, France), centrifuged at 2200 rpm (Thermo Heraeus Multifuge 1S-R) for 20 minutes without brake. To ensure the cell purity and debris exclusion, the resulting lymphocytic ring was collected, washed with PBS, resuspended in 6 ml of growing medium, and transferred in a T25 vented flask to ensure cell line establishment. This procedure was repeated three times in order to obtain three independent primed OCI-LY18 cell lines (Supplementary Figure 2).

**Supplementary Figure legends**

**Supplementary Figure 1**

Schematic representation of 3D culture model system. a) culturing systems exploited in this work — 2D: canonical cell culture; 3D ECM scaffold: human decellularized bone fragment; 3D ECM-DLBCL model: 3D ECM scaffold cultured for 72 hours with DLBCL cells (OCILY18, OCI-LY1, NU-DUL-1 or RIVA). In this 3D culture, some lymphocytes autonomously adhered to the scaffold (adherent lymphocytes), the others precipitate to the bottom of the well (non-adherent lymphocytes). The lower panels show the experimental setting of migration (b) and scaffold recellularization (c) assays.

**Supplementary Figure 2**

Schematic representation of the procedure followed for primed OCI-LY18 cell line establishment.

**Supplementary Figure 3**

Proliferation profile of OCI-LY18 cells in standard (a) and 3D (b) culture assessed by CFSE assay over a 4-day time course. CFSE mean fluorescence intensity decrease reflects the proliferation kinetics of OCI-LY18 cells.

**Supplementary Figure 4**

Annexin V analysis showing reduced apoptosis in 3D ECM-adherent OCI-LY18 and RIVA cells (a,d) upon ibrutinib treatment compared to 2D controls. MSCs co-culture does not reproduce the reduction in ibrutinib-induced apoptosis observed in ECM-adherent OCI-LY18 and RIVA cells highlighting that direct interaction with the bone-derived ECM represents the dominant driver of reduced ibrutinib sensitivity in our model (b,d) (n=3–7). Statistical analysis: ONE-WAY ANOVA with Tukey’s correction . * with respect to the non-treated controls: *p<0.05; **p<0.005; ***p<0.0005; ****p<0.0001. # with respect to the 2D treated controls: #p<0.05; ####p<0.0001. Error bars represent standard error mean.

**Supplementary Figure 5.**

Magnified view of the cytokine profiling panels shown in Figure 1. Panels b, c, and d of Figure 1 are provided here at higher resolution and enlarged scale to improve readability of cytokine labels and facilitate interpretation of the array-based results.

**Supplementary Table 1**

Evaluated cytokines. Schematic representation of the antibody printed membrane.

**Supplementary Figures**

**Supplementary Figure 1**


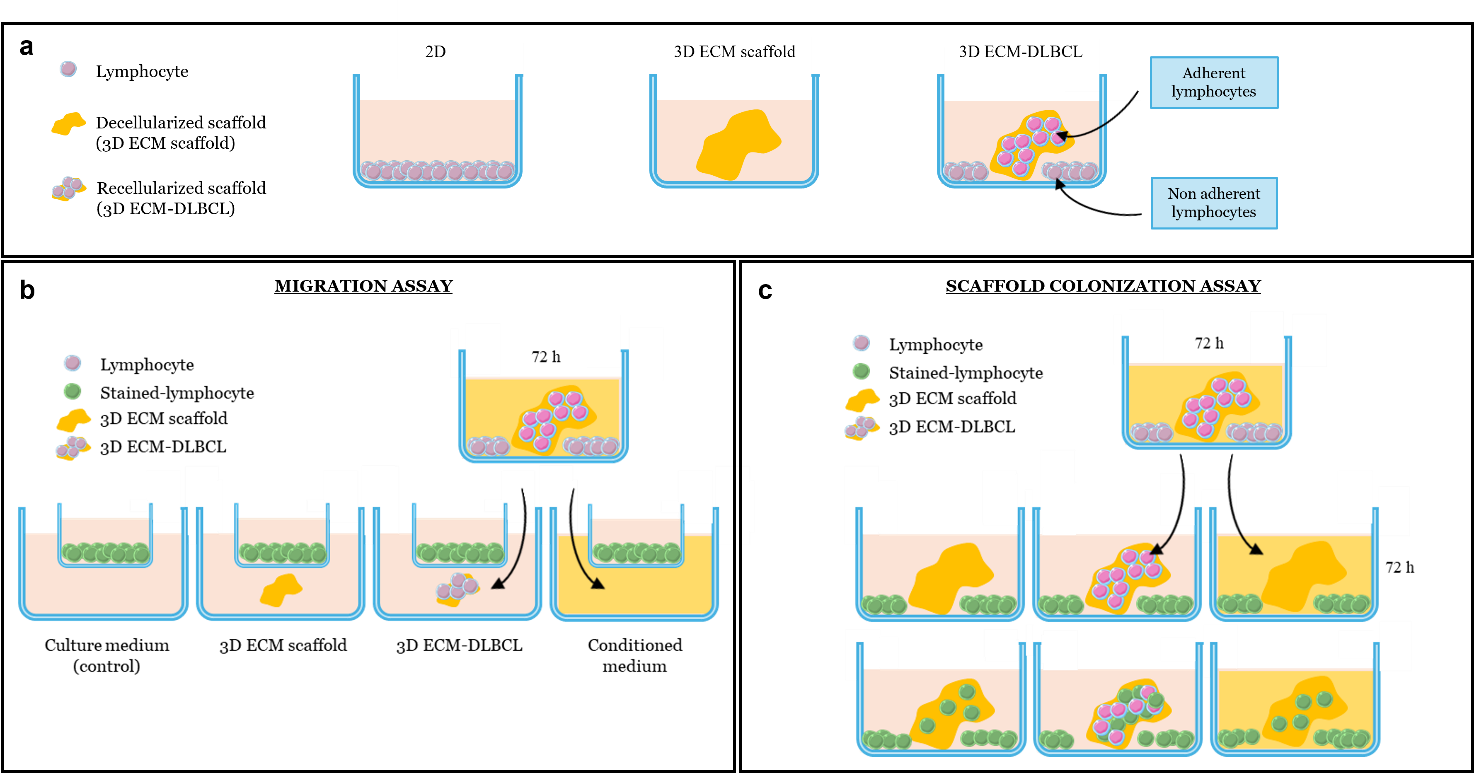


**Supplementary Figure 2**

**
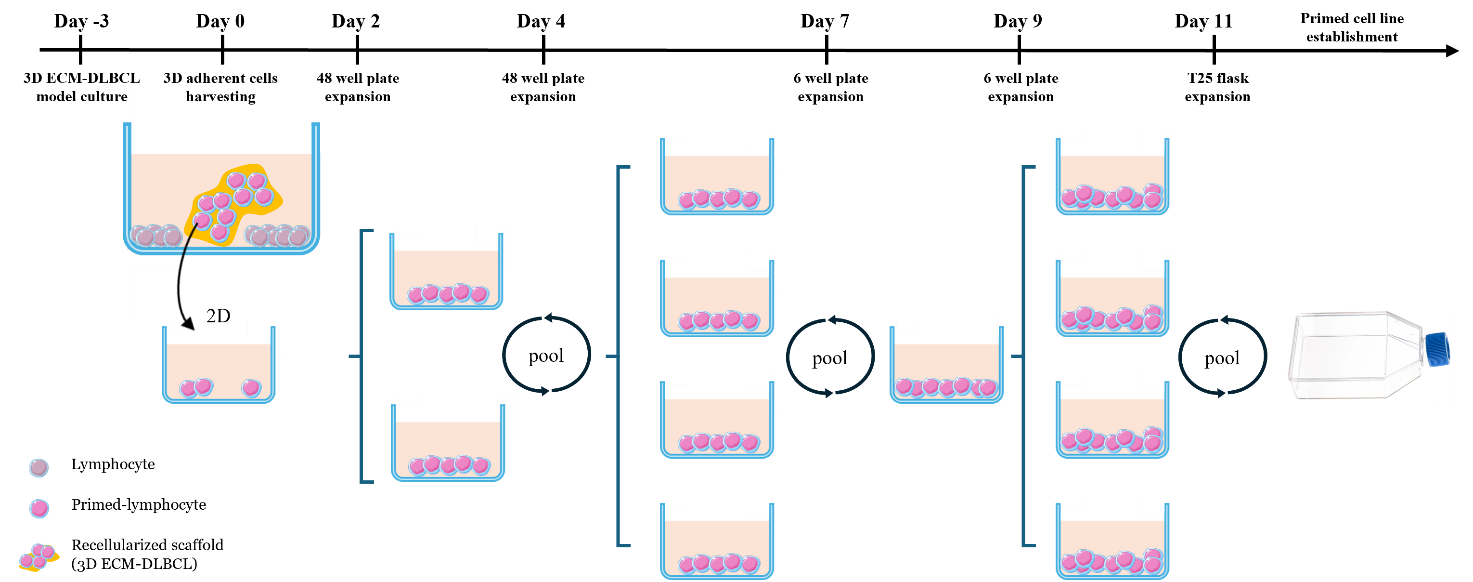
**

**Supplementary Figure 3**

**
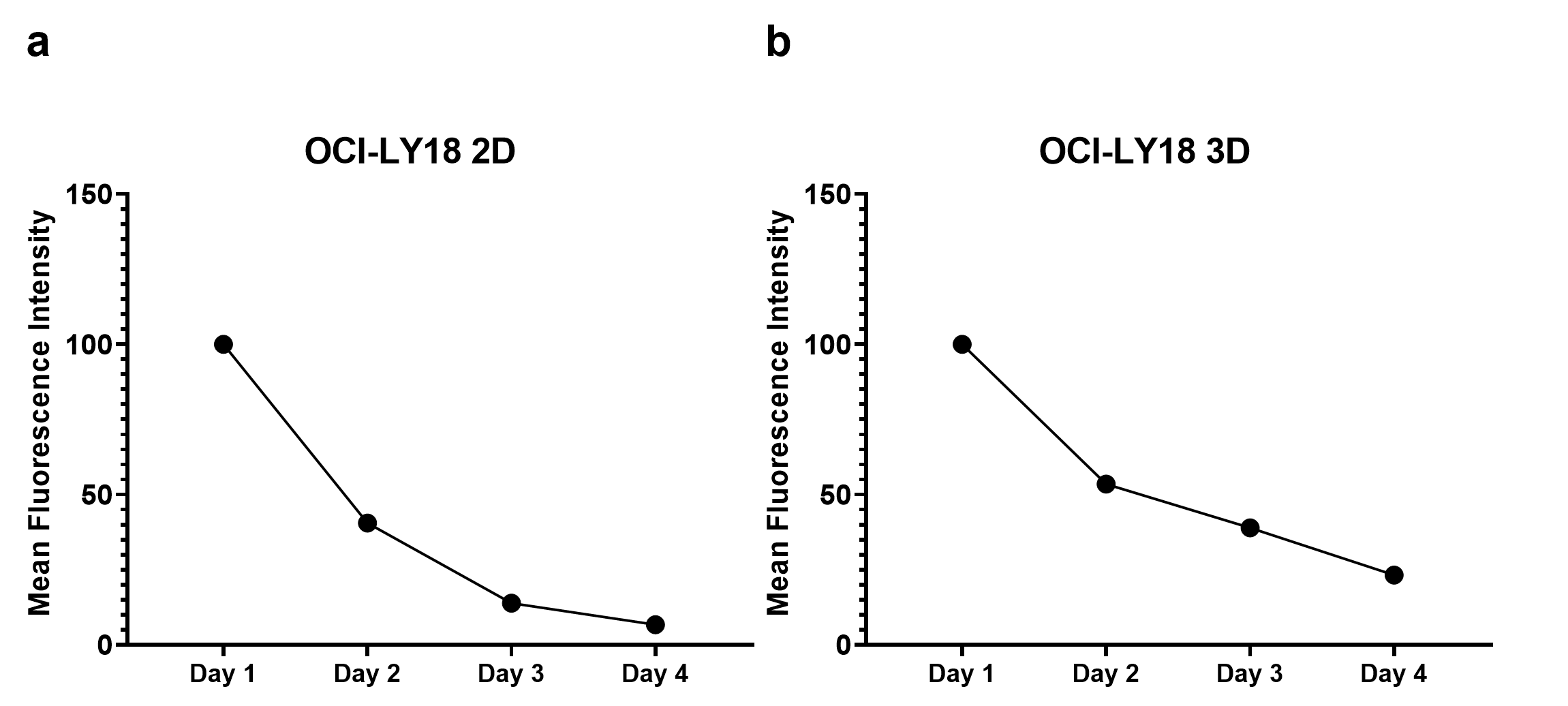
**

**Supplementary Figure 4**


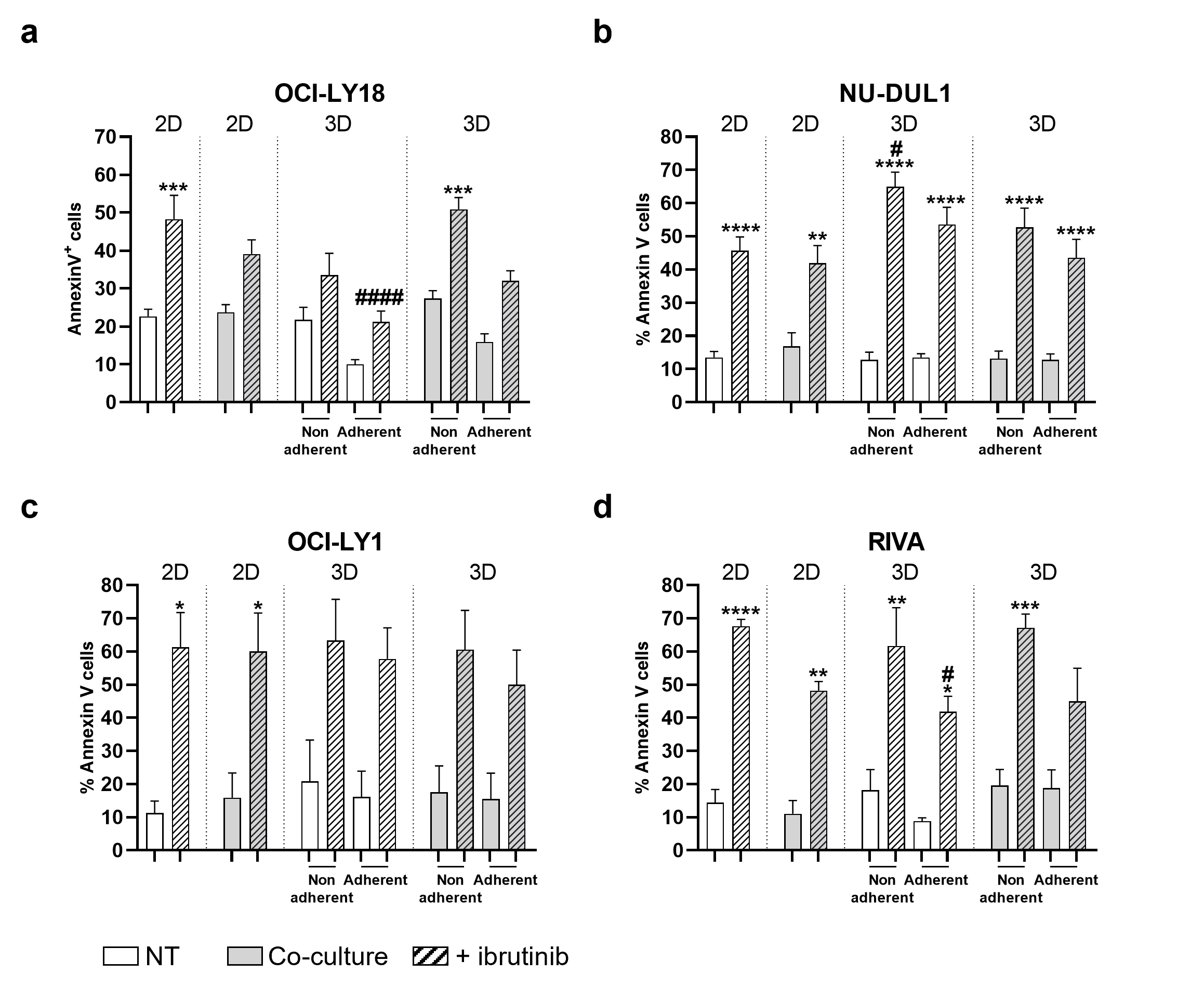


**Supplementary Figure 5**

**a**


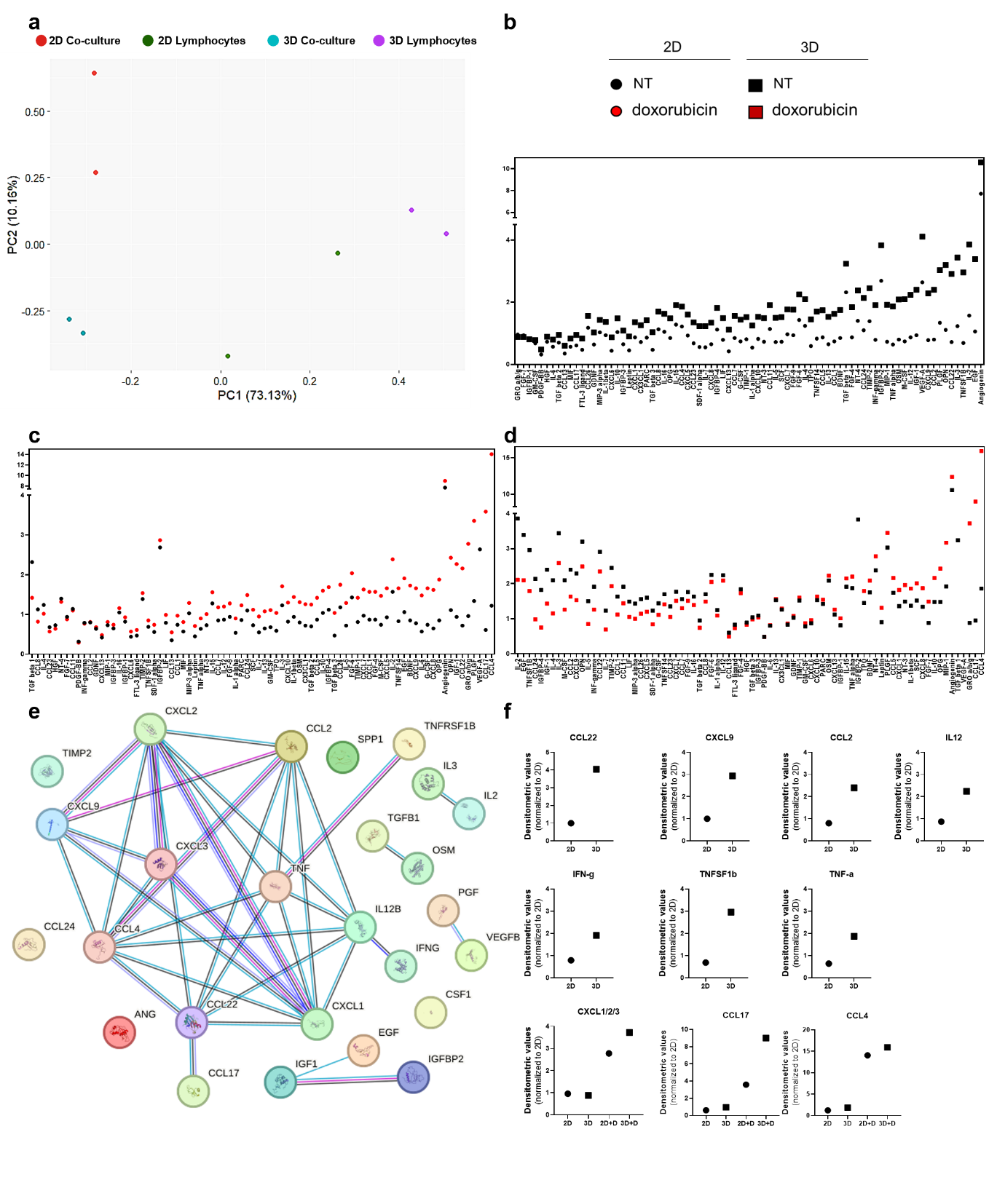


**b**


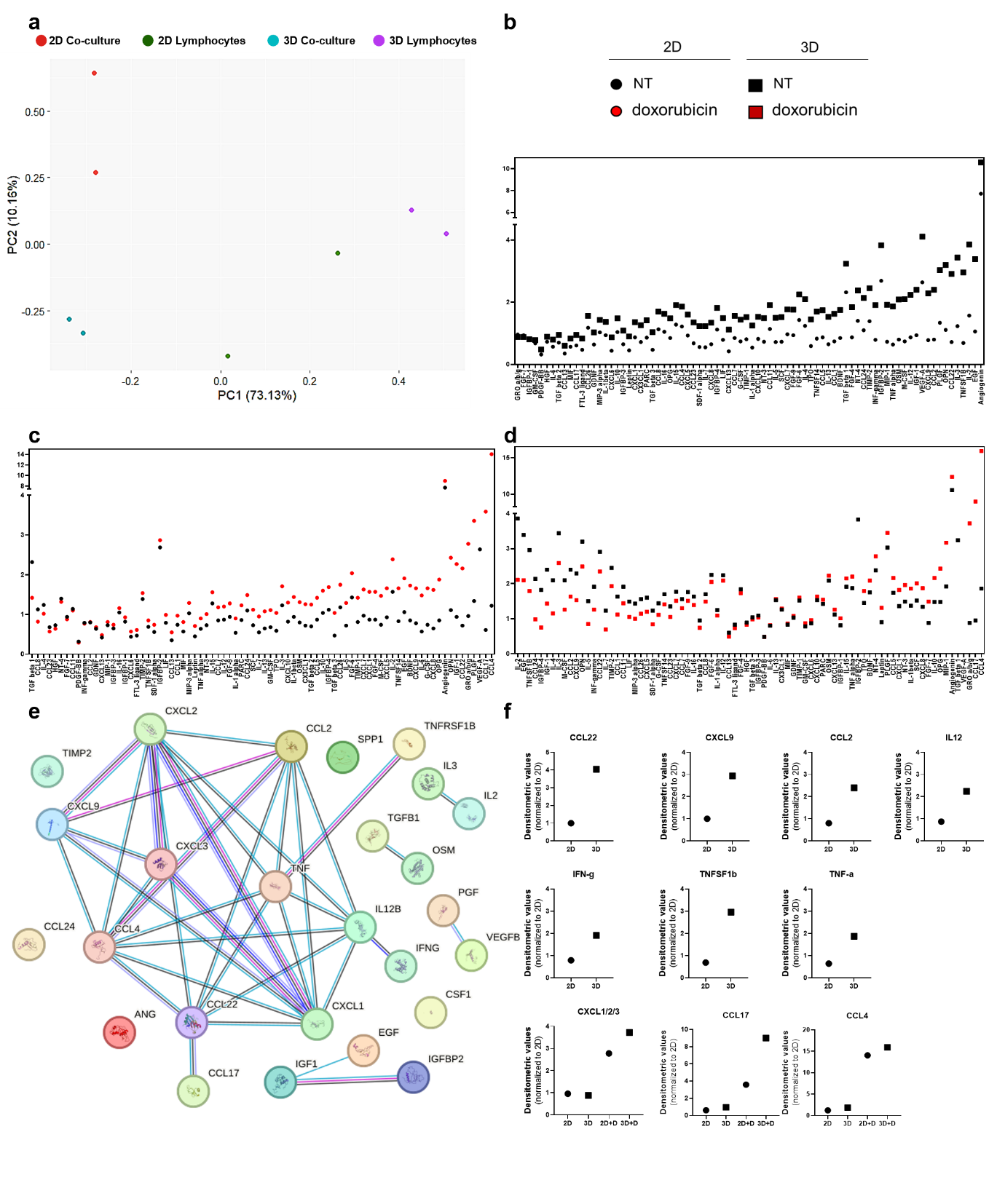


**c**


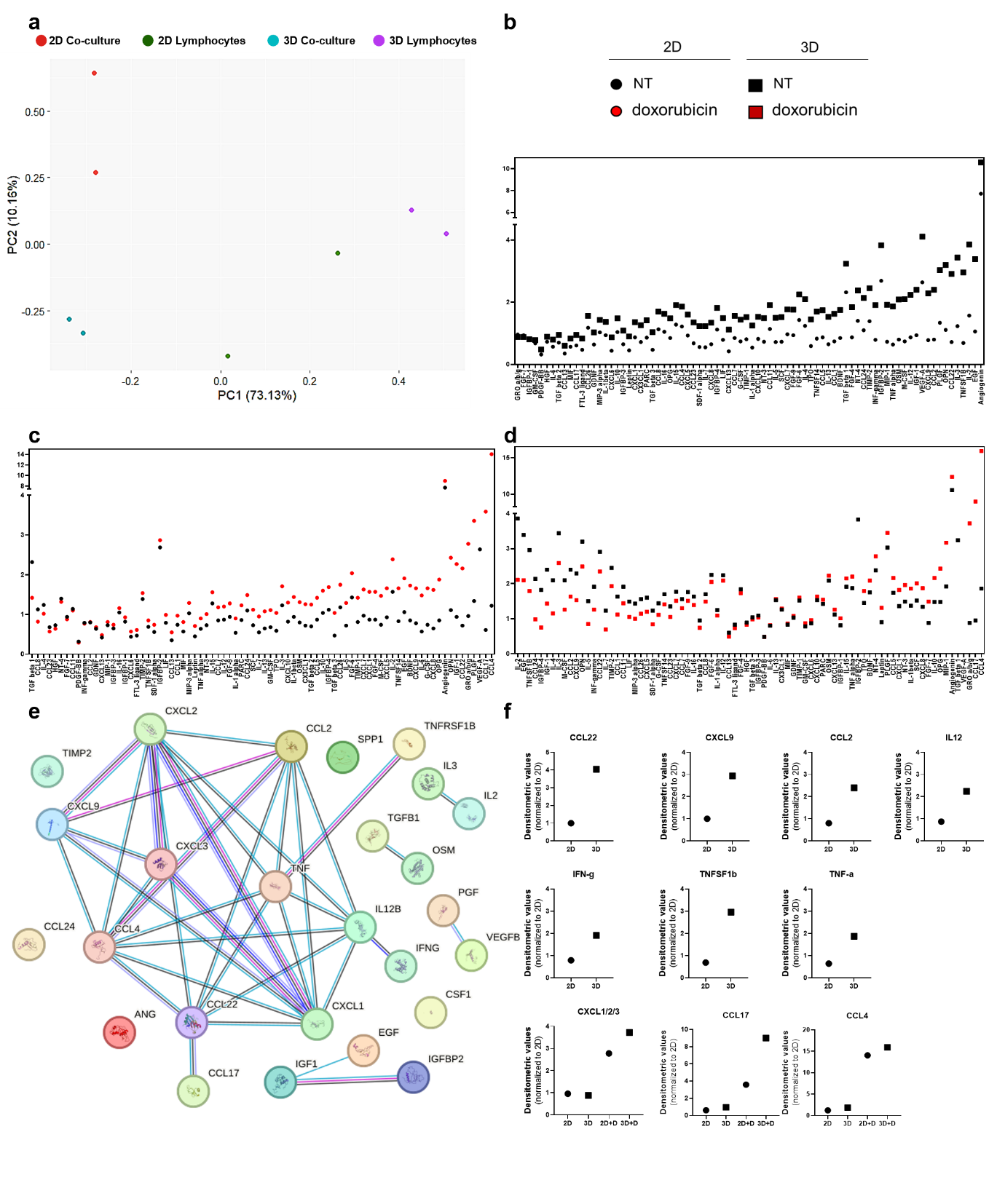


**Supplementary Table 1**


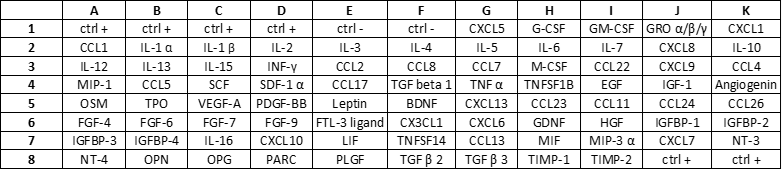

Supplement: Supplementary file 1 — Supplementary file1. [file 12032_2026_3309_MOESM1_ESM.docx]
